# Heat conjugation of antibacterial agents from amino acids and plant oil

**DOI:** 10.1038/s41598-017-11451-2

**Published:** 2017-09-07

**Authors:** Man Tang, Yanchao Zhou, Jiayang Gao, Jingli Peng, Yuan Wang, Qirui Zhao, Lihao Liao, Kai Wang, Mengjia Pan, Meng Xing, Wen Pan, Danling Dai, Min Fu, Li Yu, Chuqing Zhang, Yuchuan Wang, Ying Zhang, Li Xu, Jing Li, Xiao Bao, Wenxian Piao, Shihong Lin, Kaibei Lu, Xuelan Zhang, Weiguo Cao, Kai Yang, Zhumei He, Shaoping Weng, Qiuyun Liu, Jianguo He

**Affiliations:** 1 Guangdong Provincial Key Laboratory of Improved Variety Reproduction in Aquatic Economic Animals, State Key Laboratory of Biocontrol, Biomedical Center, Lab of Microbial Metabolic Engineering and Synthetic Biology, School of Life Sciences, Sun Yat-sen University, Guangzhou, 510275 China; 2 School of Chemistry and Chemical Engineering, Sun Yat-sen University, Guangzhou, 510275 China; 3 Division of Life Science, The Hong Kong University of Science and Technology, Clear Water Bay, Kowloon, Hong Kong; 4 Guangzhou Center for Disease Control and Prevention, Guangzhou, 510440 China; 5 Department of Genetics and Biochemistry, Clemson University, Clemson, SC 29634 USA; 6 College of Life Sciences, Jilin University, Changchun, 130012 China

## Abstract

Antimicrobial peptides are components of the innate immune systems in animals and plants as natural defense against pathogens. Critical issues like manufacturing costs have to be addressed before mass production of these peptides for agriculture or community sterilizations. Here, we report a cost-effective chemical synthesis method to produce antimicrobial cocktails, which was based on the heat conjugation of amino acids in the presence of phosphoric acid and plant oil at 150 °C. The conjugates showed potent biological activities against all tested bacteria including a multi-drug resistant *Staphylococcus aureus* strain Y5 and ampicillin resistant *Pseudomonas aerugenosa* ATCC9027 strain, demonstrating potential in agriculture, and prophylactic applications in hospital and community settings.

## Introduction

Antibiotic resistance poses great challenge to public health on a global scale^1, 2^. Drug development pipelines cannot keep up with the emergence of multi-drug resistant bacteria, and lose impetus for developing antibiotics as drug resistance quickly appears after the drugs enter market. As a potential alternative, antimicrobial peptides possessed by animals and plants are natural reservoir of defensive arsenals against pathogen^3^, to which bacteria rarely develop resistance. However, these promising antimicrobial drug candidates face the dilemma of high cost in chemical synthesis, and poor yield in fermentation productions due to toxicities to the hosts. Fatty acid conjugated antimicrobial peptides have been reported before^4, 5^, but the chemical synthesis process is either costly or technically demanding. An economical approach for antimicrobial peptide production is yet to be realized. This work presents an efficient and effective method for the synthesis of antimicrobial conjugates. The minimal inhibition concentrations of numerous products were similar to those of ampicillin against the multi-drug resistant *S*. *aureus* strain Y5 and ampicillin resistant *P*. *aerugenosa* ATCC9027 strain, revealing potential in the battle with infectious microbial agents in the foreseeable post-antibiotic era.

## Results

**Figure 1:**
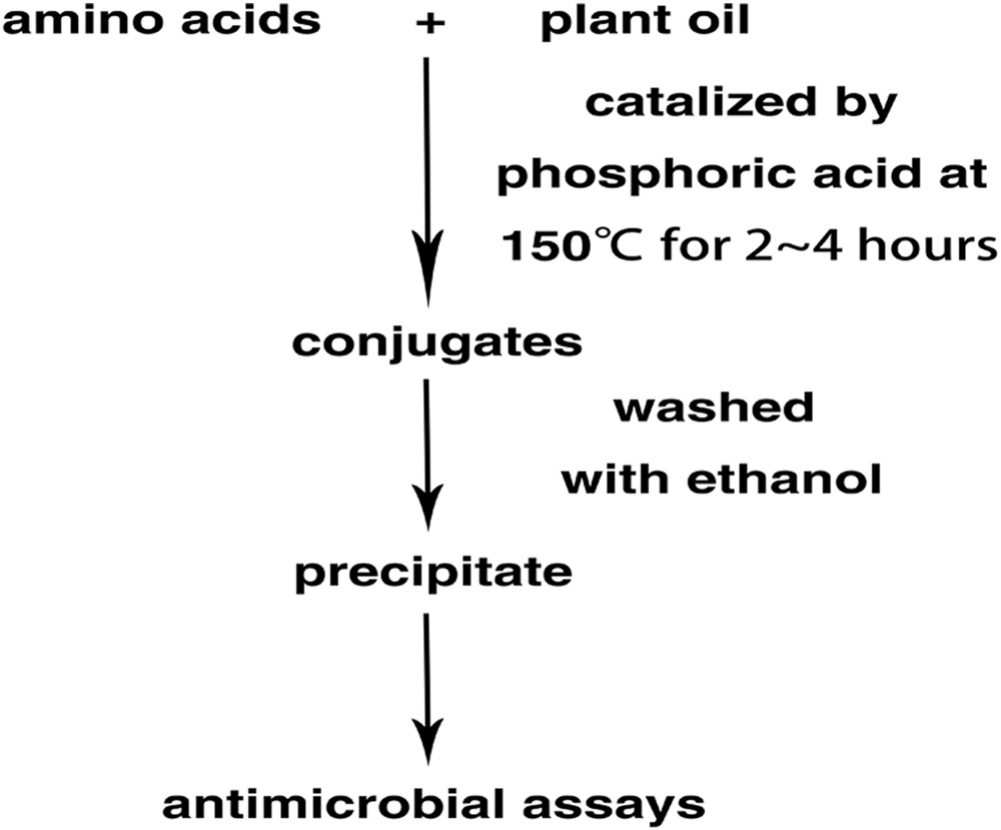
Synthesis procedure for antibacterial cocktails.

**Figure 2:**
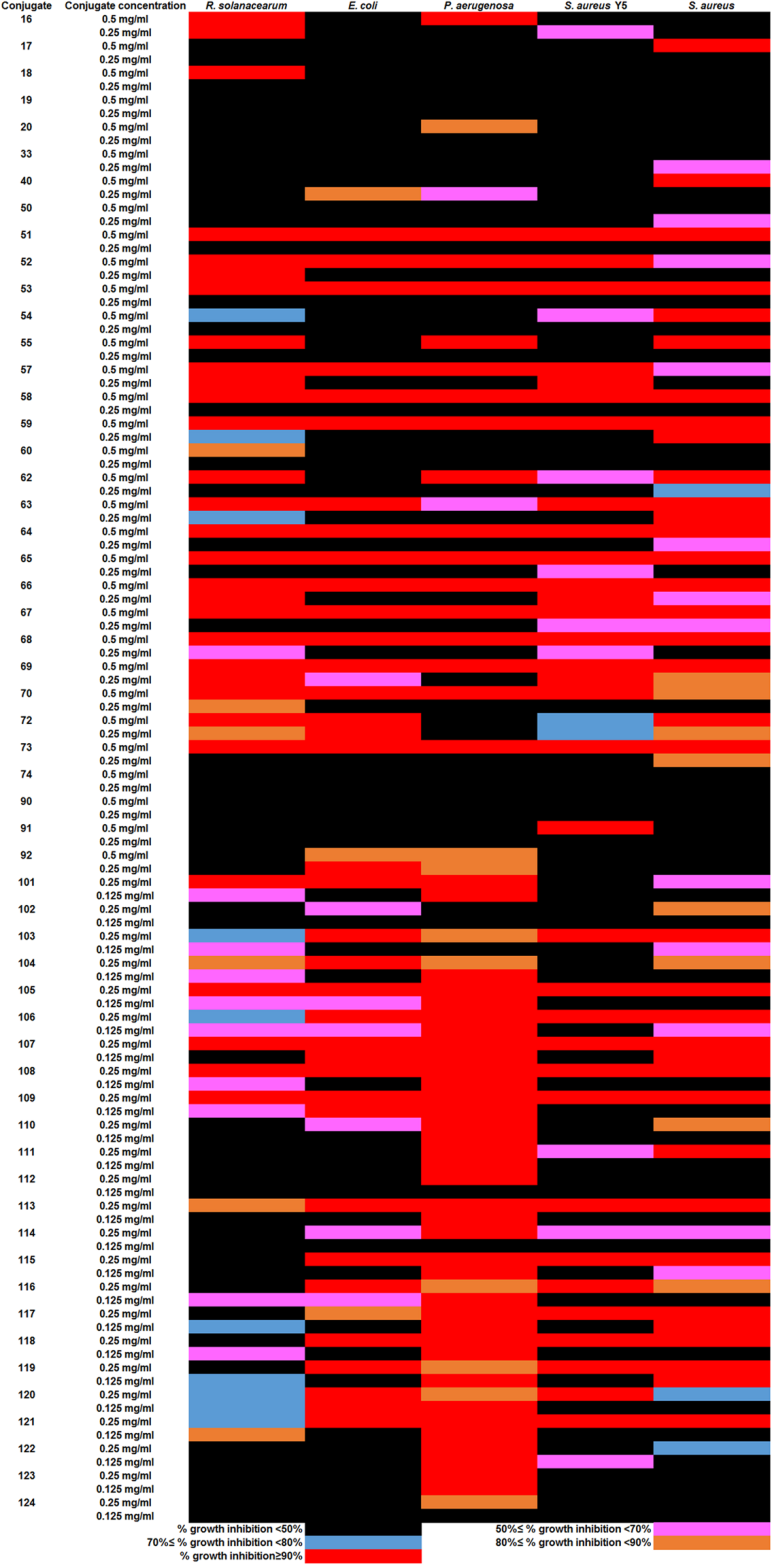
Heatmap showing that some recipes led to potent bacterial growth inhibitions in LSLP media. The conjugates numbered under 100, with at least 90% inhibitions at 0.25 mg/ml showed no lower MIC_90_ in LSLP media. The conjugates numbered over 100 with D-amino acid containing recipes displayed less than 80% inhibitions at 0.063 mg/ml in LSLP media. The MIC_90_s of ampicillin for *Ralstonia solanacearum* 1.2839, *Escherichia coli* MG1655, *P*. *aerugenosa* ATCC9027, *S*. *aureus* ATCC6538, *S*. *aureus* Y5 were as follows: 320 μg/ml, 5 μg/ml, >320 μg/ml, ≤2.5 μg/ml and 80 μg/ml in LSLP media; 160 μg/ml, 10 μg/ml, 200 μg/ml, ND (not determined) and >320 μg/ml in LB media. The MIC_90_s of kanamycin for *R*. *solanacearum* 1.2839, *E*. *coli* MG1655, *P*. *aerugenosa* ATCC9027, *S*. *aureus* ATCC6538, *S*. *aureus* Y5 were as follows: >128 μg/ml, ND, 8 μg/ml, ≤1 μg/ml and 2 μg/ml in LSLP media; >128 μg/ml, 16 μg/ml, 128 μg/ml, 32 μg/ml and 10 μg/ml in LB media.

**Figure 3:**
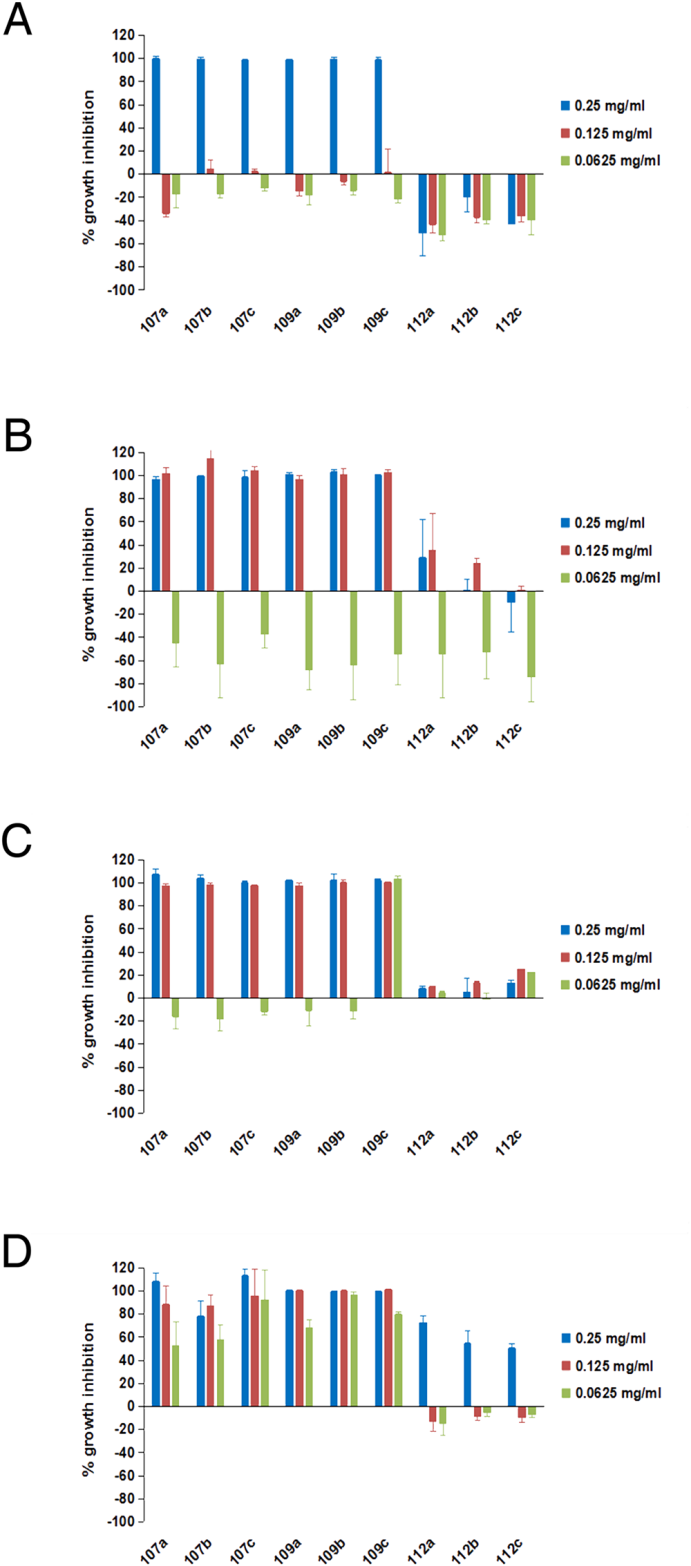
Reproducibility of antibacterial activities of the conjugates against bacteria. (**A**) *R*. *solanacearum*. (**B**) *E*. *coli*. (**C**) *P*. *aerugenosa*. (**D**) *S*. *aureus* (ATCC6538). The statistical evaluations for the reproducibility of the 3 replicates of 107 (107a, b and c) and 109 (109a, b and c) are as follows (Univariate General Linear Model repeated measures, analyzing effective concentrations with average 80% or more growth inhibitions, n = 3, 2 tailed): conjugate 107, P = 0.537 (*R*. *solanacearum*), 0.028 (*E*. *coli*), 0.031 (*P*. *aerugenosa*), 0.062 (*S*. *aureus* ATCC6538)); conjugate 109: P = 0.824 (*R*. *solanacearum*), 0.222 (*E*. *coli*), 0.527 (*P*. *aerugenosa*), 0.725 (*S*. *aureus* ATCC6538, Greenhouse-Geisser correction). Assays for each strain were performed in triplicate in 0.5 X LSLP media (1 X LSLP media for *R*. *solanacearum*), and average values are shown with one standard deviation.

**Figure 4:**
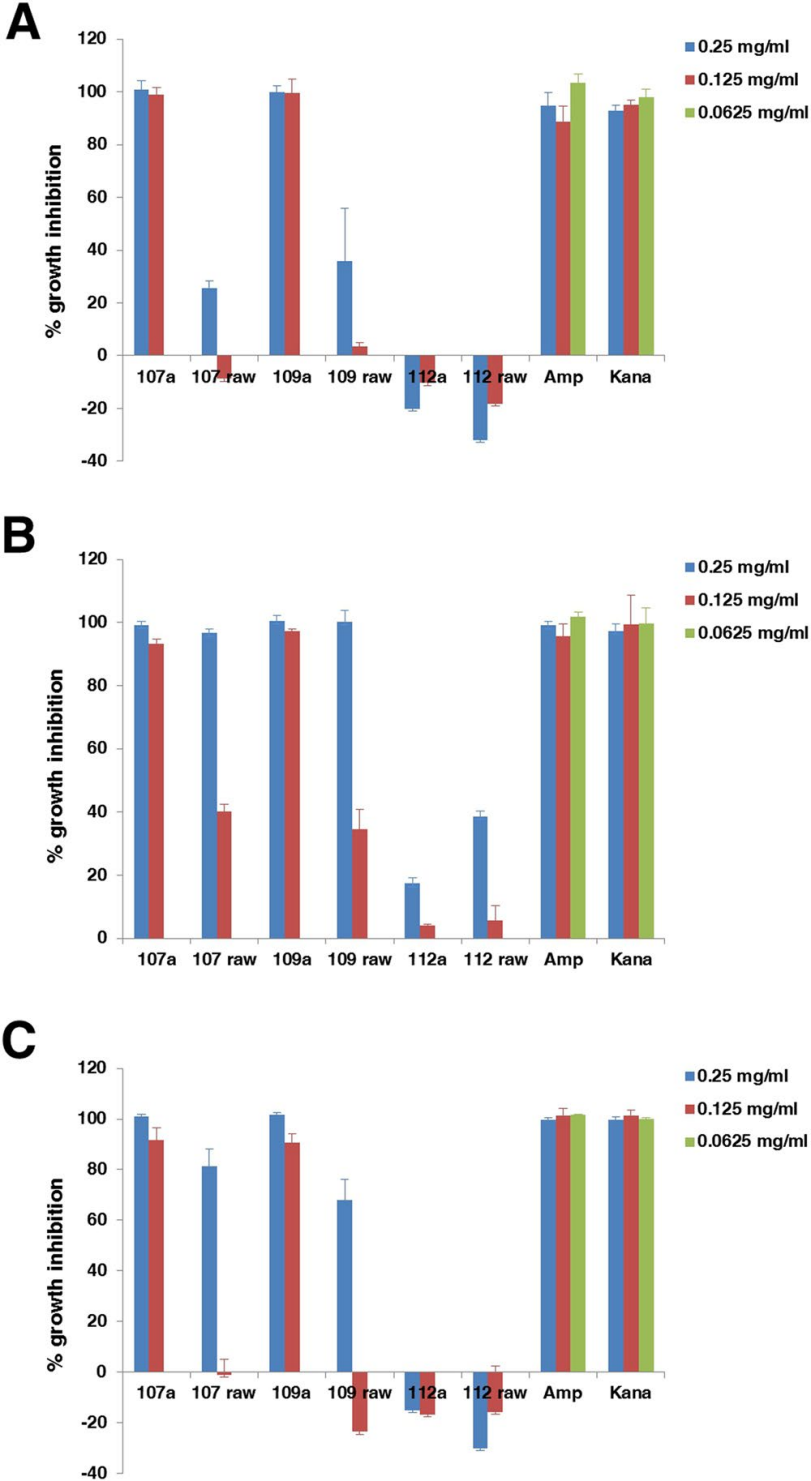
Percent growth inhibitions of raw materials against bacteria. P = 0.000 for 107a and 109a conjugate samples versus corresponding raw material samples against *E*. *coli* MG1655 (**A**), *S*. *aureus* (ATCC6538) (**B**), *S*. *aureus* Y5 (**C**) respectively (Univariate General Linear Model, n = 3, 2 tailed). Assays for each strain were performed in triplicate in 0.5 X LSLP media, and average values are shown with one standard deviation. Amp: ampicillin. Kana: Kanamycin.

We first established the procedure and successfully synthesized antibacterial cocktails using different amino acid recipes (Fig. 1, Supplementary Tables S1 and S2). The yields of the conjugation reactions were over 30% based on the amount of total raw materials used (Table 1). Electron microscopy examinations showed that the *P*. *aerugenosa* cell structure treated with conjugate 107 was irregular (Supplementary Fig. S1B) while all the rest of the cells after treatment remained intact (Supplementary Fig. S1), which is different from the observations made on various conventional antimicrobial peptides. Unlike large number of naturally occurring antimicrobial peptides, membrane permeability upon treatments with the conjugates was virtually unchanged (Supplementary Fig. S2), suggesting that a distinct mechanism underlies such phenomena. The conjugated products 107 and 109 were efficacious in the presence of anions of strong acids, oxalate and acetate, and they were more effective in the presence of calcium salts than some sodium salts despite that some calcium salts were insufficiently soluble (Supplementary Fig. S3). Growth susceptibility assays indicated that some recipes led to potent biological activities in LSLP (Low Salt and Low Protein) media (Fig. 2) and Low Protein media (Supplementary Fig. S4), and activities were not attenuated by the inclusion of 1% NaCl in the Low Protein media. Although the conjugates were slightly acidic due to the use of phosphoric acid as catalyst (Supplementary Table S1), most control bacteria grew well at pH as low as 4.0 in LSLP media (Supplementary Fig. S5). Antibacterial activities were reduced at high protein media such as standard LB (Supplementary Fig. S6). The 10 fold reduction in protein and peptide concentration in the LSLP media from LB media resulted in approximately 10 fold reduction of the MICs (Fig. 2, Supplementary Figs S4 and S6). The 3 replicates of the most active conjugates 107 and 109 demonstrated good reproducibility respectively with small variations of activities when they were examined in parallel (Fig. 3). The control conjugates 112 showed virtual absences of antibacterial activities, and they even substantially enhanced the growth of one of the four types of bacteria tested. The conjugates 107 and 109 possessed more potent activities against three types of bacteria tested than its corresponding raw materials (Fig. 4). The raw material of control 112 showed absence of antibacterial activities. Mass spectral analyses demonstrated that the conjugates were mixtures with different mass/charge ratios (Supplementary Figs S7–S15). The signature compounds of conjugate 107 with an *m/z* 127.8–127.9, 163.8 and 256.8 are predicted to be 5-pyrrolidone-2-carboxylic acid, phenylalanine, and lysine, N-(5-oxoprolyl) (Supplementary Figs S7–S9). The signature compounds of conjugate 109 with an *m/z* 115.9,143.9 and 341.9 are projected to be proline, N-methyl-5-pyrrolidone-2-carboxylic acid and (P/E/P) tri-peptide with undefined amino acid sequence order (Supplementary Figs S10–S12). One of the signature compounds of conjugate 109 with an *m/z* 226.9 owned a likely proline, 5-oxoprolyl structure (Supplementary Figs S10–S12). A common feature shared between conjugates 107 and 109 is that they both possessed molecules with proline-like moieties, probably accounting for their consistency in antibacterial activities. The signature compounds of conjugate 112 with an *m/z* 178.9–179.0,192.8–193.0,194.2 are predicted to be gluconolactone, SS dipeptide, and 2-amino-3-(1,2-dihydroxyethyl) succinic acid. None of the signature compounds harbored fatty acid or phosphoric acid moieties.

**Table 1:** Yield of the thermal conjugation reactions with 3 replicates for each recipe*.

Conjugate no.	Raw materials			Weight of products (g)	Yield based on total raw materials used
	Amino acids (g)	H_3_PO_4_ (μl)	Peanut oil (ml)		
107a	3.000	600	4.800	2.945	35.9%
107b	3.000	600	4.800	2.873	35.0%
107c	3.000	600	4.800	3.051	37.2%
109a	3.000	600	4.800	2.561	31.2%
109b	3.000	600	4.800	2.973	36.3%
109c	3.000	600	4.800	2.852	34.8%
112a	3.000	600	4.800	3.121	38.1%
112b	3.000	600	4.800	3.102	37.8%
112c	3.000	600	4.800	2.852	34.8%

^*^The weight of 600.0 μl H_3_PO_4_ and 4.800 ml Peanut oil were 1.151 g and 4.048 g respectively.

^1^H Nuclear Magnetic Resonance (NMR) spectra of hydrolysates of the conjugates indicated that some of the chemical shifts can be attributed to methine, methylene, and methyl protons (Supplementary Figs S16–S24). The absorption at 4–5 ppm can be assigned to α-carbon protons of amino acid residues^6, 7^. The absorption at 8–10 ppm demonstrated the presence of branched and/or opened amide groups^6–8^. The NMR data showed that the hydrolysates had complex molecular compositions and are difficult to categorize (Supplementary Table S3 and Supplementary Figs S16–S24). The extracted oil and amino acid contents in the hydrolysates were reproducible among duplicate samples (Supplementary Table S4), and oil contents were substantially different before and after n-hexane extractions on the conjugates. Ninhydrin assays with strong light absorbance at 440 nm for measuring proline and hydroxyproline suggest that the aforementioned prediction of structurally similar 5-pyrrolidone-2-carboxylic acid and lysine, N-(5-oxoprolyl) in conjugate 107 is accurate, in which no proline was used as raw material. Dynamic Light Scattering (DLS) examinations showed that the particle sizes of the conjugates were heterogeneous (Supplementary Fig. S25).

## Discussion

Although antimicrobial peptides have shown promise in preclinical studies, its potential has not been fully tapped yet. Our approach demonstrates the advantages of the development of inexpensive and available antibacterial agents suitable for many practical applications that cannot be tackled with classical antimicrobial peptides due to their high production cost. For instance, the agents can be used for various topical applications that do not require FDA approval, in clinics as sterilization agent, as components for bacteriostatic soaps, or in agriculture.

It has been reported that the heat conjugation of aspartic acid could be performed with sulfolane and phosphoric acid^8^. However, sulfolane is toxic to human body and is also an environmental pollutant. The plant oil we used as solvent is safe and economical in industrial production. With environmentally friendly raw materials used in the process, this approach provides a green chemistry solution to persistent public health and agricultural issues.

The antimicrobial conjugates may have trapped various ions such as anions of strong acids or organic acids for its bactericidal or bacteriostatic activities when bacteria cell walls and cytoplasmic membranes remained intact during treatments. The local buildup of some ions may have interfered the functions of some molecules and obstruct the operation of crucial cellular machinery. The insoluble rigid salts formed between divalent cations and organic acids may damage bacteria cells as well. Numerous organic acids have modest median lethal doses on animals. For instance, compounds with similar structures to oxalate, such as ethanol and acetic acid, could extend lifespans^9, 10^, probably by inhibiting oxalate generation as it is toxic to cells. In contrast, most conventional antimicrobial peptides act against bacteria via membrane disruption^11^. Naturally occurring antimicrobial peptides also hamper DNA and protein synthesis, protein folding, and cell wall synthesis. The amphipathicity of the conventional antimicrobial peptides allow them to insert into membrane bilayers to form pores by “barrel-stave”, “carpet”, “toroidal-pore” mechanisms. They can also enter cells to target intracellular molecules or components which are vital to cell survival. Given the presence of ionic bonding and secondary chemical bonding in proteins and peptides, ion trafficking might have changed in protein and peptide rich LB media, attenuating the activities of antibacterial conjugates by impeding the local or intracellular buildup of some ions. It suggests that the conjugated products could be best used in low protein environments such as disease prevention in agriculture, sterilizations in hospital and community settings. With little proteins present in the surfaces of plant structures, antibacterial conjugates may execute the bacteriostatic effects at low concentrations. *R*. *solanacearum* is a plant pathogen, and the projected concentrations for field use against the bacterium are below 100 μg/ml. The MIC_90_s of the most active conjugates 107 and 109 against the multi-drug resistant *S*. *aureus* strain Y5 were low in 1/2 X LSLP media, suggesting that the use of the inexpensive conjugates for large scale sterilizations of drug resistant bacteria is feasible. The distribution of mass/charge ratios suggests that the heat conjugates are chemical cocktails with complex compositions. Phosphoric acid was involved in the catalysis and formation of peptide bonds as both a catalyst and part of intermediates in previous reports^12–14^, which accounts for the modest acidity of the conjugation products in this study. Yet, equal amounts of raw materials 107 and 109 possessed lower activities than the conjugates respectively, and the antibacterial activities of control raw material 112 were virtually absent. The unrestricted accessibility of raw materials should find widespread use of this method in the fight against various disease-causing bacteria in the wake of widespread emergence of antibiotics resistant microorganisms. Antifungal, antiviral and anti-insect conjugates can be synthesized in a similar fashion, and characterizations are underway.

## Methods

### Bacterial strains and reagents

*R*. *solanacearum* (1.2839) was obtained from China General Microbiological Culture Collection Center, CGMCC. The multi-drug resistant *S*. *aureus* Y5 strain was previously described^10^. *E*. *coli* MG1655 was provided by the *E*. *coli* Genetic Stock Center. *S*. *aureus* (ATCC6538) and *P*. *aerugenosa* (ATCC9027) was obtained from Guangdong Microbiology Culture Center. High pure amino acids were provided by BBI (BBI Life Sciences Corporation, HK, China). Peanut Oil was manufactured by Guangdong COFCO Food Sales & Distribution Co., Ltd (Guangdong, China). Arowana Sunflower Seed Oil was provided by the Yihai Kerry Kellogg Foods (Shanghai) Co., Ltd. (Shanghai, China). Luhua Pressed Corn Oil and Sesame Oil were products of Shandong Luhua Fragrant Peanut Oil Co., Ltd. (Shandong, China). The Pure Pressed Rapeseed Oil was manufactured by Deyang Nianfeng Food Co., Ltd. (Deyang, Sichuan, China). Phosphoric acid (≥85%) was provided by the Fuyu Fine Chemical Co., Ltd. (Tianjin, China). The recipe for LSLP media is as follows: 0.05% yeast extract, 0.1% trytone and 0.0001% NaCl. The recipe for Low Protein media is the same as LSLP media except the supplement of 1% NaCl. 1/2 X LSLP media is half the strength of LSLP media.

### Bacterial susceptibility assays

Bacterial susceptibility assays were determined according to reference^5^. Briefly, 50 µl of 1 × 10^6^/ml bacterial cells was mixed with 50 µl conjugates with 2 fold serial dilutions. Growth inhibitions were recorded via measuring the absorbance at 492 nm in a Thermo Scientific Multiskan FC microplate reader (Thermo Fisher Scientific, Waltham, MA) after 20 h of incubations at 30 °C (*R*. *solanacearum* 1.2839) or 37 °C (rest). The assays for each concentrations of each sample were performed in triplicate. Microbial Inhibition halos were generated as described^15^, and 10^6^ cfu/ml bacterial cells were plated for detection of antimicrobial activities.

### Conjugation method for antimicrobial cocktails

Amino acids were mixed with and submerged in plant oil and phosphoric acid, and plant oil and phosphoric acid were added at a ratio of 8:1 (v/v). Accordingly, 3 g of amino acids were mixed with 0.6 ml phosphoric acid and 4.8 ml peanut oil. After heat conjugation at 150 °C for 4 h, the sticky and warm mixtures were washed with 3 volumes of absolute ethanol for 2 to 3 times, and centrifuged at 6,676 × g for 10 min. Precipitates were stored at 4 °C for future use. The average costs for amino acids, peanut oil and phosphoric acid were around 7 $/KG, 2 $/KG and 2 $/KG in China respectively, and the manufacturing cost was about 1 $/KG, for a total of about 12 $/KG or less depending on the prices of raw materials.

### N-hexane extraction of antibacterial conjugates

30 mg conjugates were mixed with 9 volumes of n-hexane for extraction of oil for 3 h at 55 °C at 200 rpm^16^. Supernatant was dried at 50 °C. The same amount of chloroform as n-hexane was added to the precipitate for extraction for 3 h at 55 °C at 200 rpm. Supernatant and precipitates were dried at 50 °C respectively and weighted. pH values of the dried substances were determined at 0.02 mg/ml.

### Hydrolysis and analysis of antibacterial conjugates

100 μl 0.2 M NaOH was added to 20 mg conjugates, which was incubated at 28 °C for 2.5 h. 60 μl absolute ethanol was added and dried at 50 °C. Subsequently 50 μl absolute ethanol was added and dried at 50 °C. The last step was repeated once. Hydrolysates were mixed with 9 volumes (initial conjugate equivalent) of n-hexane for extraction for 3 h at 55 °C at 200 rpm. Supernatant was dried at 50 °C. Amino acids in residuals were determined by Ninhydrin assays. Ninhydrin Amino Acid Indicator solution included the following reagents: 0.5 g ninhydrin, 0.3 g fructose, 22.68 g Na_2_HPO_4_, 6 g KH_2_PO_4_ in a total volume of 100 ml. 570 μl samples or standard solutions were mixed with 30 μl Ninhydrin Amino Acid Indicator solution. Boiling for 15 min was performed after mixing well. Light absorbance was determined at 440 nm (for proline and hydroxyproline) and 570 nm (other amino acids). Amino acid mixtures listed in the recipes of antimicrobial conjugates 107, 109 and 112 were used for standard curve calibrations respectively. Hydrolysates have been finally subjected to ^1^H NMR analysis.

### Transmission electron microscopy of bacterial cells

Samples containing bacterial cells in PBS were incubated with conjugates at 30 °C (*R*. *solanacearum*) or 37 °C (rest) at concentration of 1 mg/ml. Controls containing bacterial cells in PBS were incubated without conjugates at the same temperature. Subsequently, all samples were centrifuged at 7,300× g for 1 min, and washed with PBS twice followed by centrifugation. A drop containing the bacteria was absorbed onto a copper grid and negatively stained with phosphotungstic acid for 30 s. The grids were examined using a JEM-100CX-II electron microscope (JEOL Ltd., Japan)^17^.

### Membrane permeability assays

ONPG (o-nitrophenyl-β-D-galactopyranoiside) can cross cytoplasmic membrane when membrane permeability has changed, which is then subjected to hydrolysis by β-galactosidase. A yellow substance o-nitrophenol released can be measured at 420 nm in a Thermo Scientific Multiskan FC Microplate Photometer (Thermo Fisher Scientific, Waltham, MA). Log phase *E*. *coli* ML-35 was washed 3 times and resuspended in 10 mM sodium phosphate buffer (pH 7.4 with 0.1 M NaCl). Peptide solutions and 1.5 mM ONPG (final concentration) prepared in 10 mM sodium phosphate buffer, and 1 × 10^7^ cfu of bacteria were loaded in each well in 96-well microtitre dishes, and A420 was measured at different time points. Data were reported as absorbance values minus negative control values with conjugates^4^.

### Liquid Chromatography-Mass Spectrometry

Conjugates were dissolved in water. Subsequently Liquid Chromatography-Mass Spectrometry was performed on TSQ Quantum UltraTM (Mass Spectrometer (Thermo Fisher Scientific, San Jose, CA, USA).

### Dynamic Light Scattering (DLS)

6 mg/ml of conjugates dissolved in water were used for DLS measurements at 20 °C on a BI-200SM goniometer (Brookhaven Instruments, Holtzville, NY, USA).

### NMR

A solution of the hydrolysate of a specific conjugate in D_2_O (600 μl) was added to a dry NMR tube. The ^1^H NMR spectra were collected on Varian INOVA 500NB NMR Spectrometer (Varian Inc., Palo Alto, CA, USA) at ambient temperature.

### Statistical analyses

All statistical analyses were performed using SPSS 22.0. The experiments were evaluated using Descriptive, the General Linear Model Univariate or Repeated Measures. Parametric tests were conducted when data were approximately normal distributed after examining with Shapiro-Wilk tests or Kolmogorov-Smirnov tests. The alpha level for all tests was 0.05.

### Data Availability

All data generated or analyzed during this study are included in this published article and its Supplementary Information file.

## Electronic supplementary material

SUPPLEMENTARY INFORMATION
