# Supplementary material for: Heat conjugation of antibacterial agents from amino acids and plant oil
Source: Sci Rep. 2017 Sep 7;7:10852. doi: 10.1038/s41598-017-11451-2 (PMC5589812; doi:10.1038/s41598-017-11451-2)
Supplement: Supplementary file 1 — SUPPLEMENTARY INFORMATION [file 41598_2017_11451_MOESM1_ESM.pdf]

Supplementary Information

Heat conjugation of antibacterial agents from amino acids and plant oil

Man Tang[1*], Yanchao Zhou[1*], Jiayang Gao[1*], Jingli Peng[1*], Yuan Wang[1*], Qirui Zhao[1*], Lihao Liao[2], Kai Wang[1,3], Mengjia Pan[1], Meng Xing[1], Wen Pan[1], Danling Dai[1], Min Fu[1], Li Yu[1], Chuqing Zhang[1], Yuchuan Wang[2], Ying Zhang[4], Li Xu[1], Jing Li[1,5†], Xiao Bao[1], Wenxian Piao[1], Shihong Lin[1], Kaibei Lu[1], Xuelan Zhang[6], Weiguo Cao[1,5], Kai Yang[1], Zhumei He[1], Shaoping Weng[1], Qiuyun Liu[1] & Jianguo He[1]

Affiliations:

[1]Guangdong Provincial Key Laboratory of Improved Variety Reproduction in Aquatic Economic Animals, State Key Laboratory of Biocontrol, Biomedical Center, Lab of Microbial Metabolic Engineering and Synthetic Biology, School of Life Sciences, Sun Yat-sen University, Guangzhou 510275, China. [2]School of Chemistry and Chemical Engineering, Sun Yat-sen University, Guangzhou 510275, China. [3]Division of Life Science, The Hong Kong University of Science and Technology, Clear Water Bay, Kowloon, Hong Kong. [4]Guangzhou Center for Disease Control and Prevention, Guangzhou 510440, China. [5]Department of Genetics and Biochemistry, Clemson University, Clemson, SC 29634, USA. [6]College of Life Sciences, Jilin University, Changchun 130012, China. [*]These authors contributed equally to this work. Correspondence and requests for materials should be addressed to Q.L. (lsslqy@mail. sysu. edu. cn) or to J.H. (lsshjg@mail. sysu. edu. cn)

Tel. : 86-20-84110296; Fax: 86-20-84036551;

School of Life Sciences, Sun Yat-Sen University, Guangzhou 510275, P. R. China.

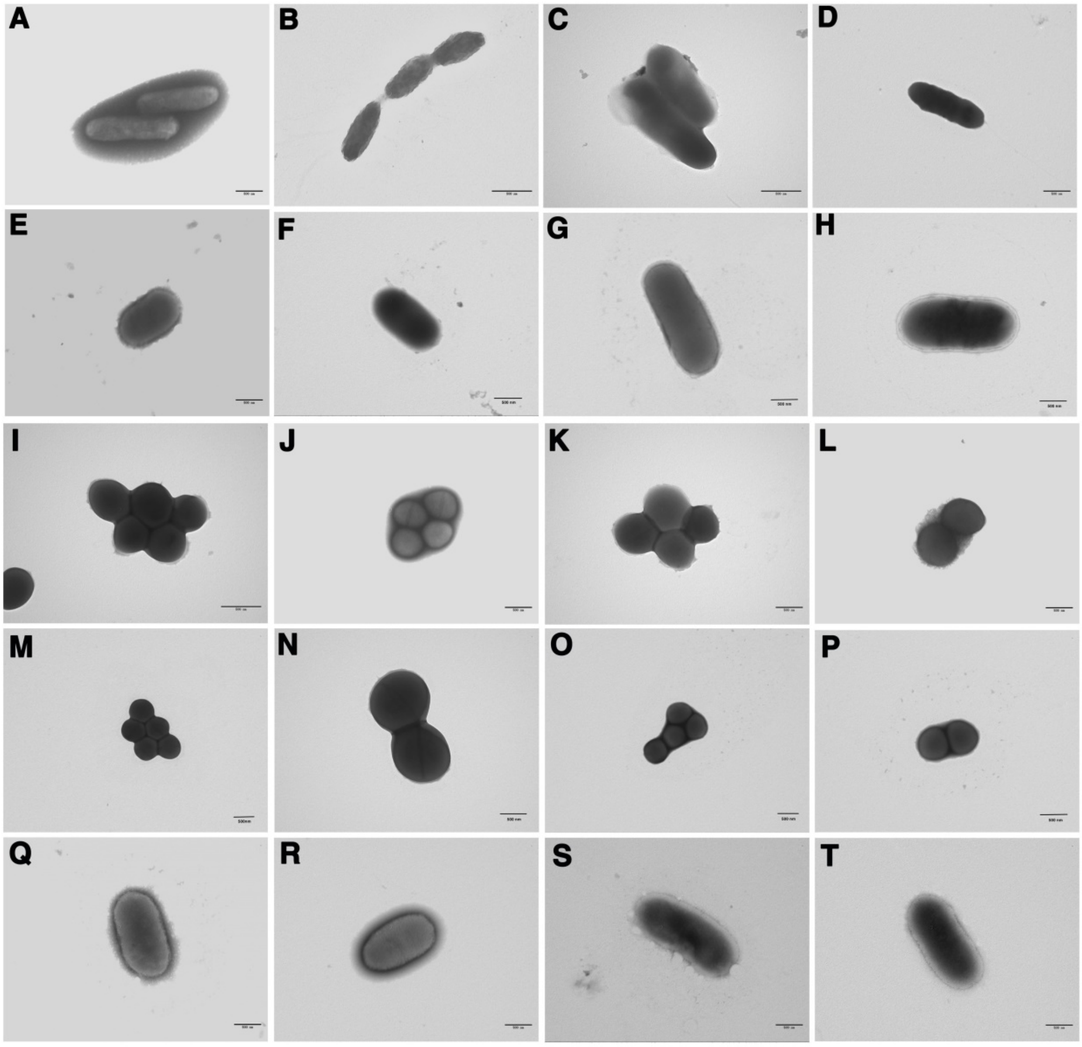

Supplementary Fig. S1 Electron microscopy on antibacterial activities. (A-D) *P. aerugenosa* cells: Control , 1 mg/ml conjugate 107, 1 mg/ml conjugate 109, 1 mg/ml control conjugate 112. Rests of the samples are ordered likewise. (E-H) *E. coli* MG1655. (I-L) *S. aureus* ATCC6538. (M-P) *S. aureus* Y5. (Q-T) *R. solanacearum* 1.2839. Scale bar: 500nm.

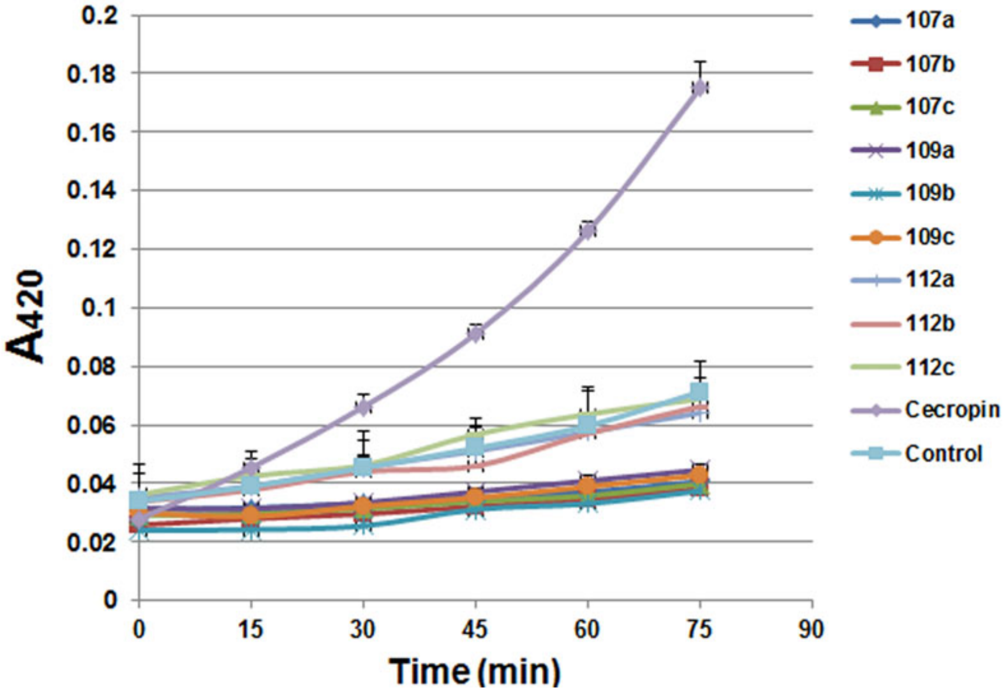

Supplementary Fig. S2 Membrane permeability assays with different antibacterial conjugates. Assays for each time point of each sample were performed in triplicate, and average values are shown with one standard deviation. 1 mg/ml conjugates were used in each well, and 10 μM cecropin A was present in the positive control.

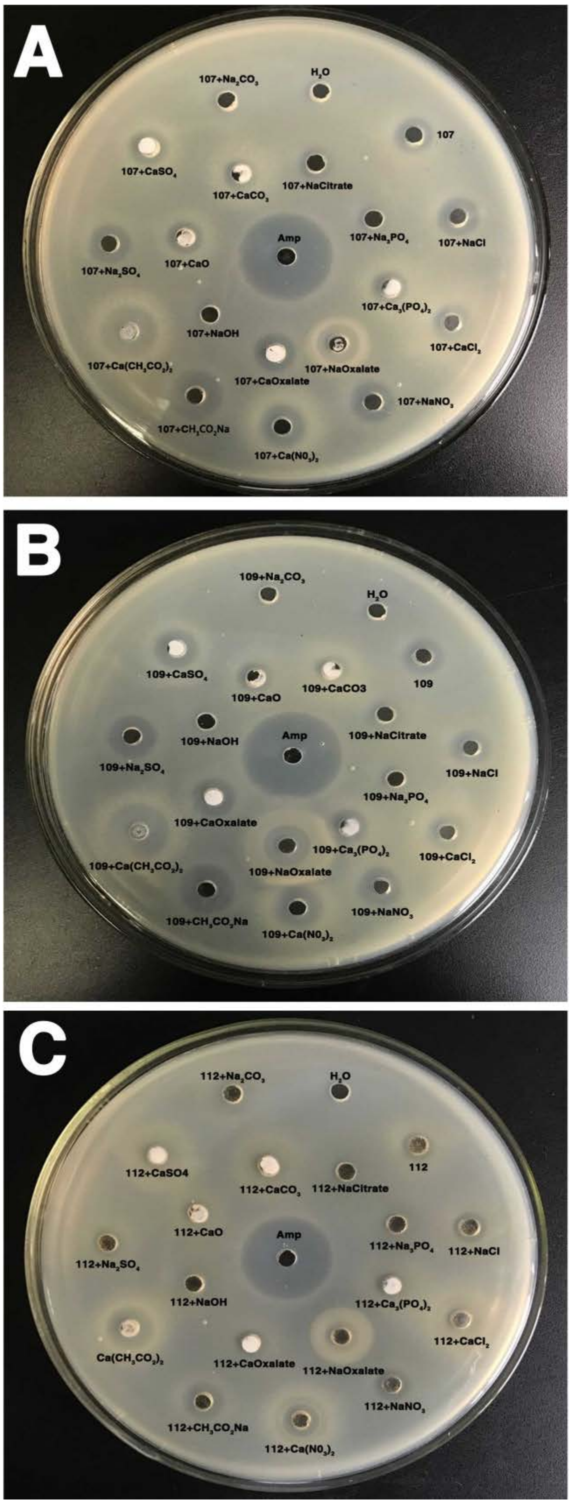

Supplementary Fig. S3 Inhibition halos generated by the use of antibacterial conjugates against *E. coli* MG1655 in the presence of different salts or substances. 1 mg NaCl was used in a well, and other salts of equivalent normality were loaded per well for comparisons. Each well contained 2 mg antibacterial conjugates. (A) Conjugate 107. (B) Conjugate 109. (C) control Conjugate 112. The punched wells were 0.5 cm in diameter. Photograph was taken after 14 h incubation at 37 °C.

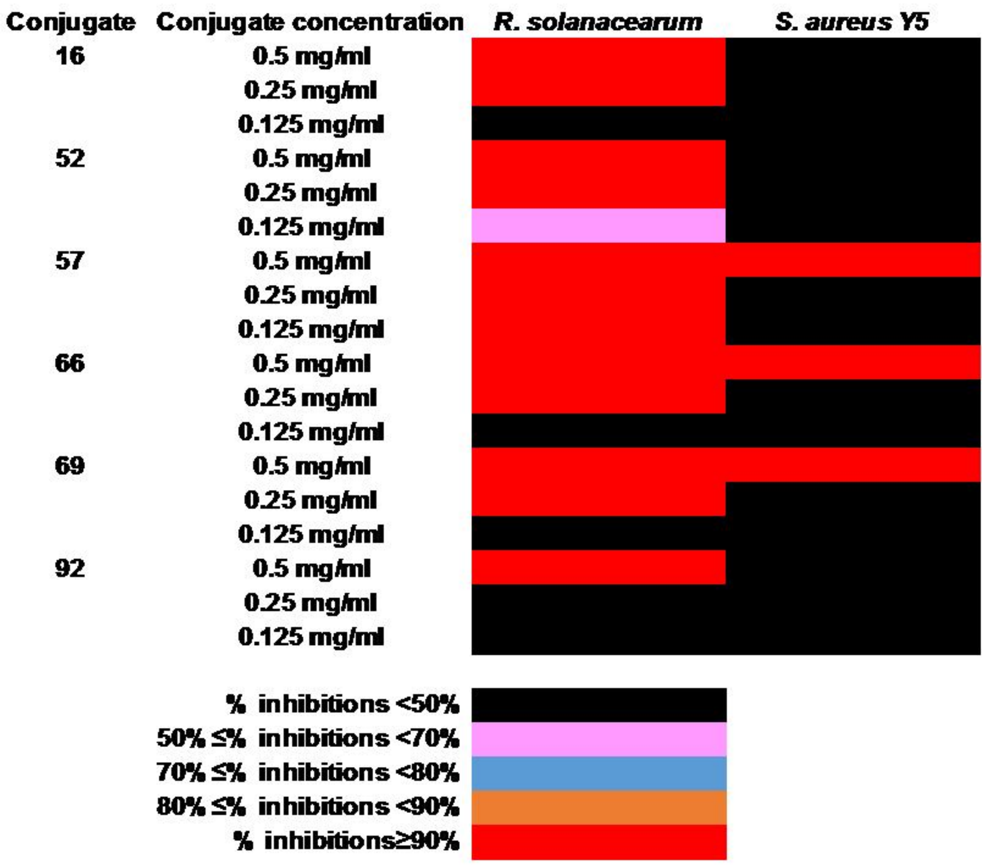

Supplementary Fig. S4 Heatmap showing that some recipes led to potent bacterial growth inhibitions in Low Protein media with 1% NaCl.

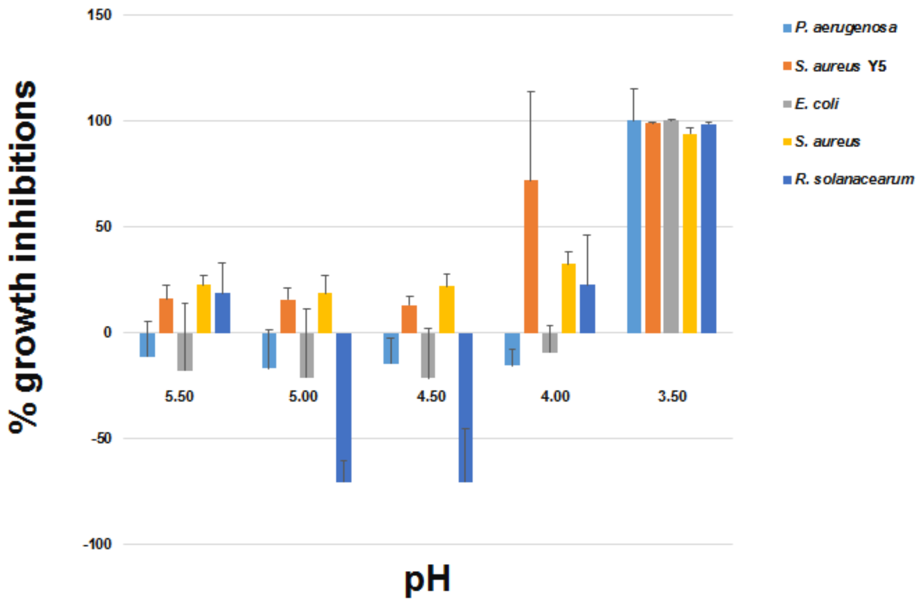

Supplementary Fig. S5 Bacterial growth in LSLP media at different pH. pH were adjusted with dilute HCl. Assays for each strain at each pH values were performed in triplicate, and average values are shown with one standard deviation.

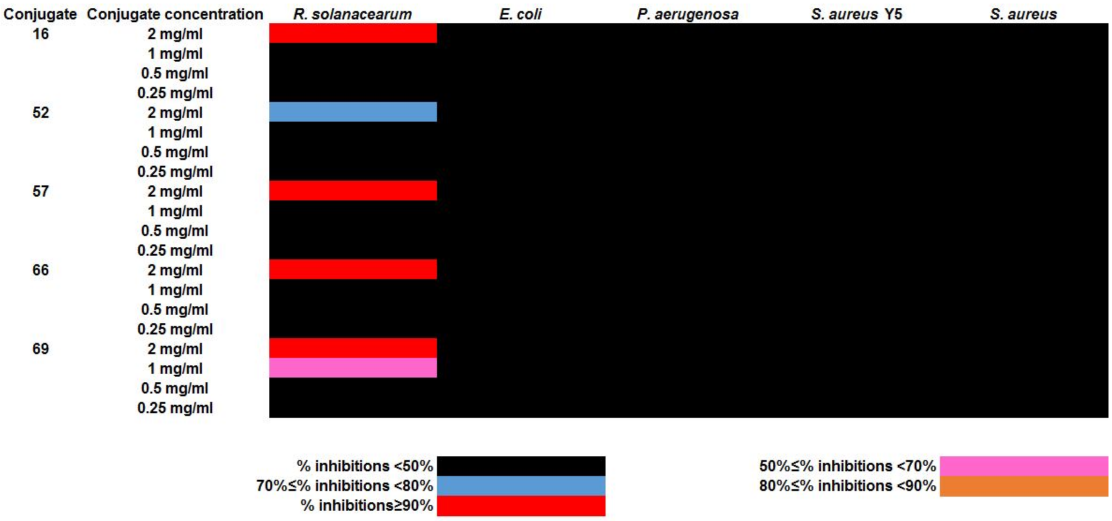

Supplementary Fig. S6 Antibacterial activities measured by percent growth inhibitions were reduced at high protein media Luria Broth (LB).

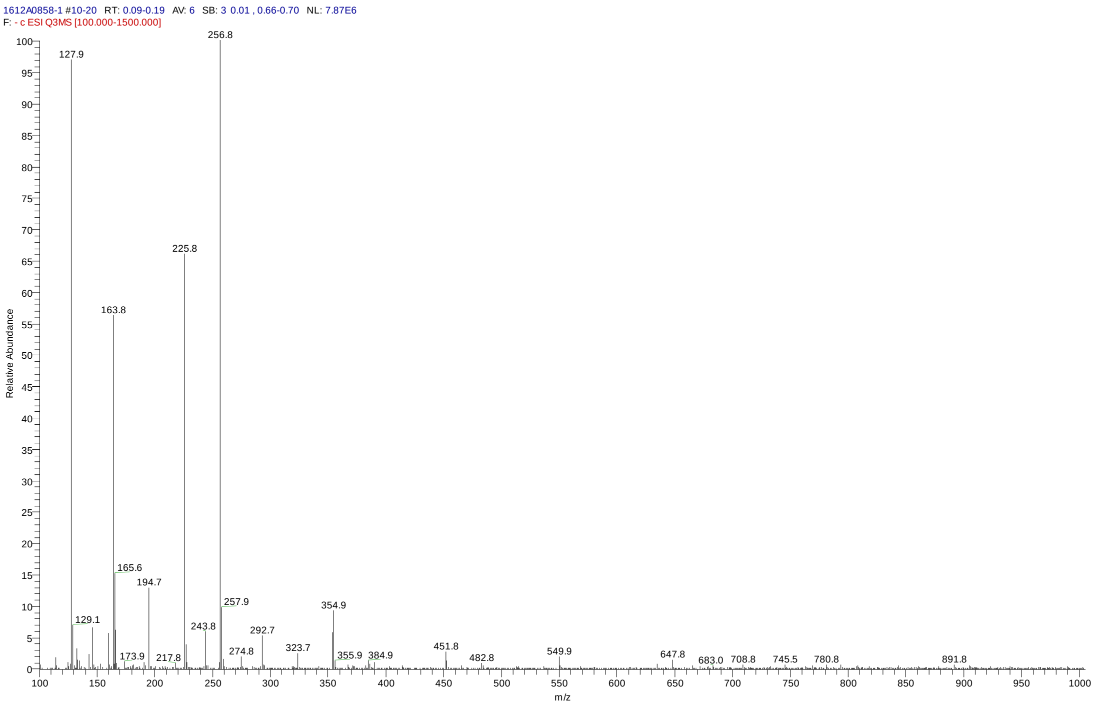

Supplementary Fig. S7 Mass spectral analysis of amino acid conjugate 107a. Vertical coordinate: relative abundance. Horizontal coordinate: m/z.

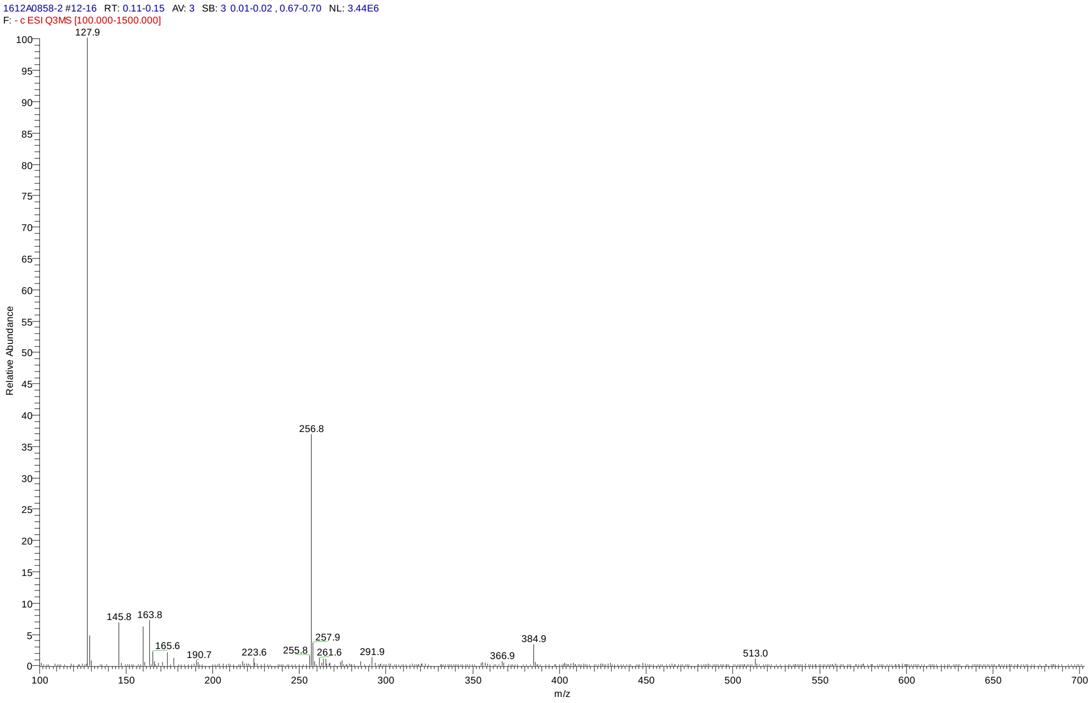

Supplementary Fig. S8 Mass spectral analysis of amino acid conjugate 107b.

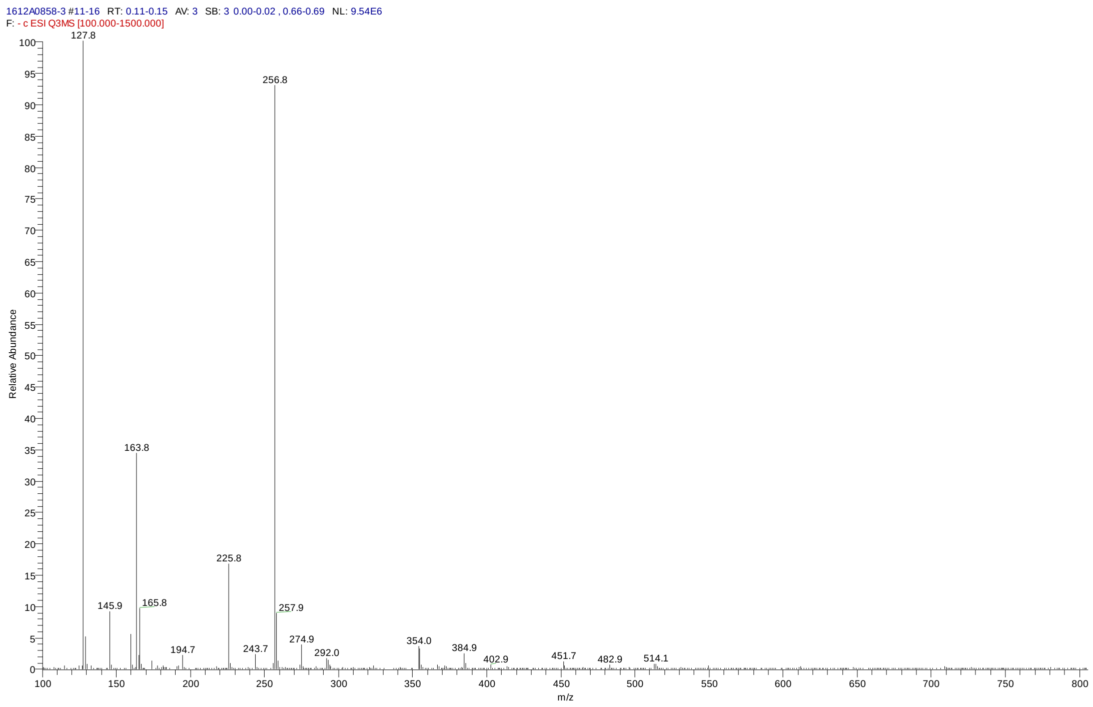

Supplementary Fig. S9 Mass spectral analysis of amino acid conjugate 107c.

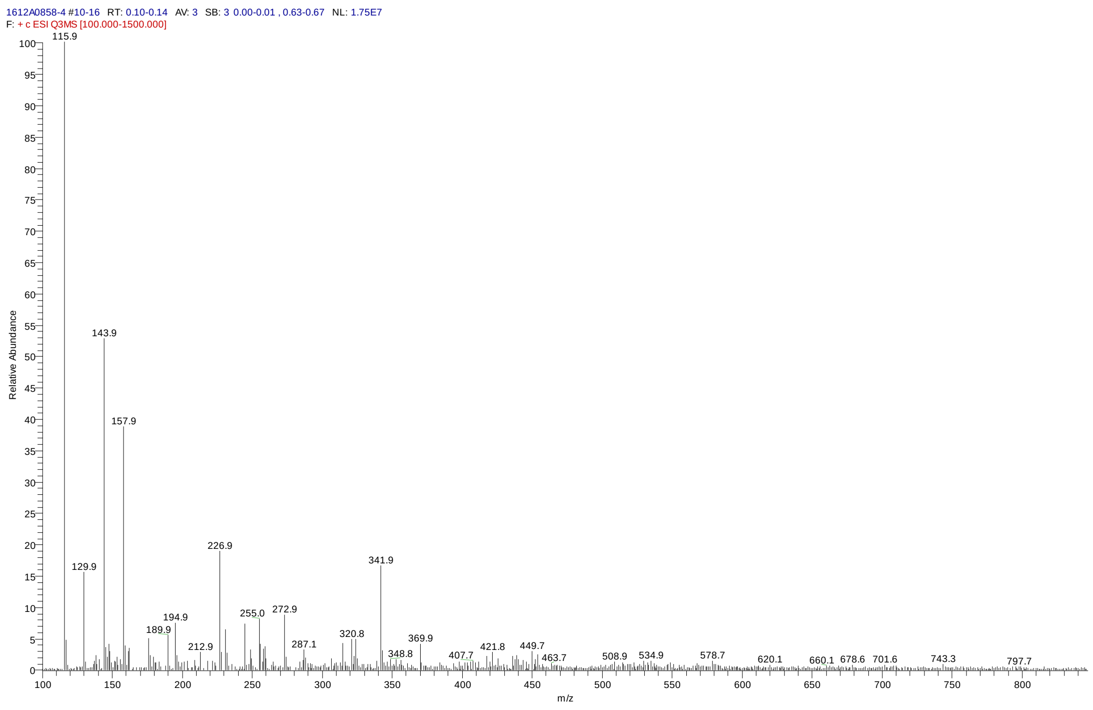

Supplementary Fig. S10 Mass spectral analysis of amino acid conjugate 109a.

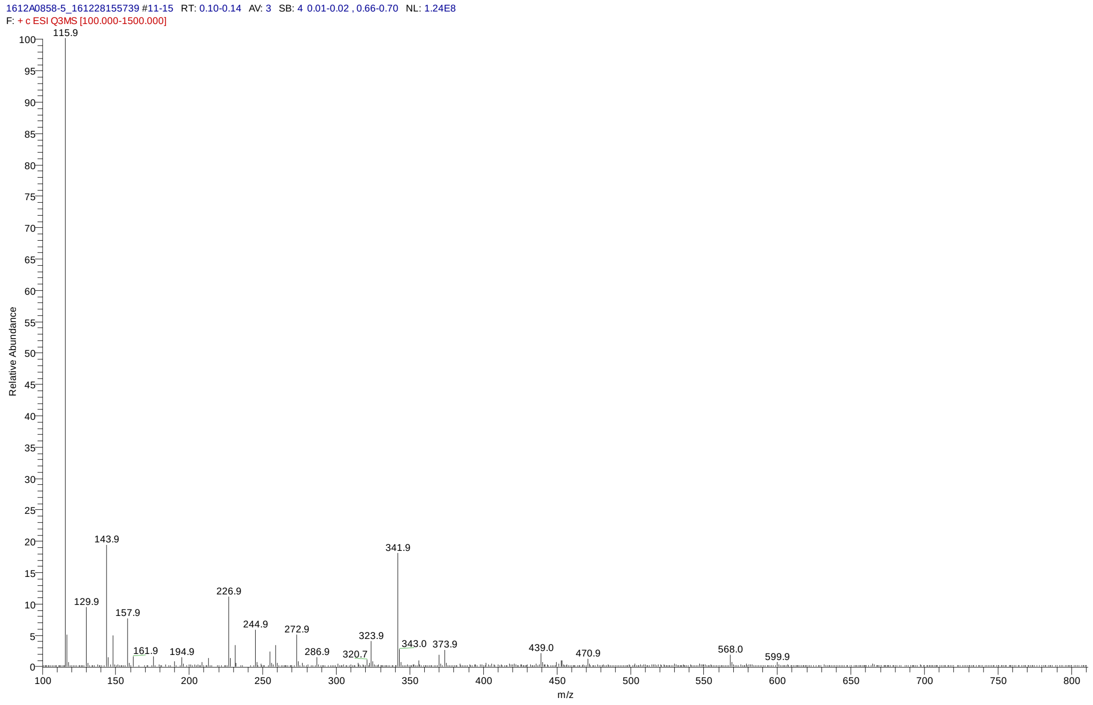

Supplementary Fig. S11 Mass spectral analysis of amino acid conjugate 109b.

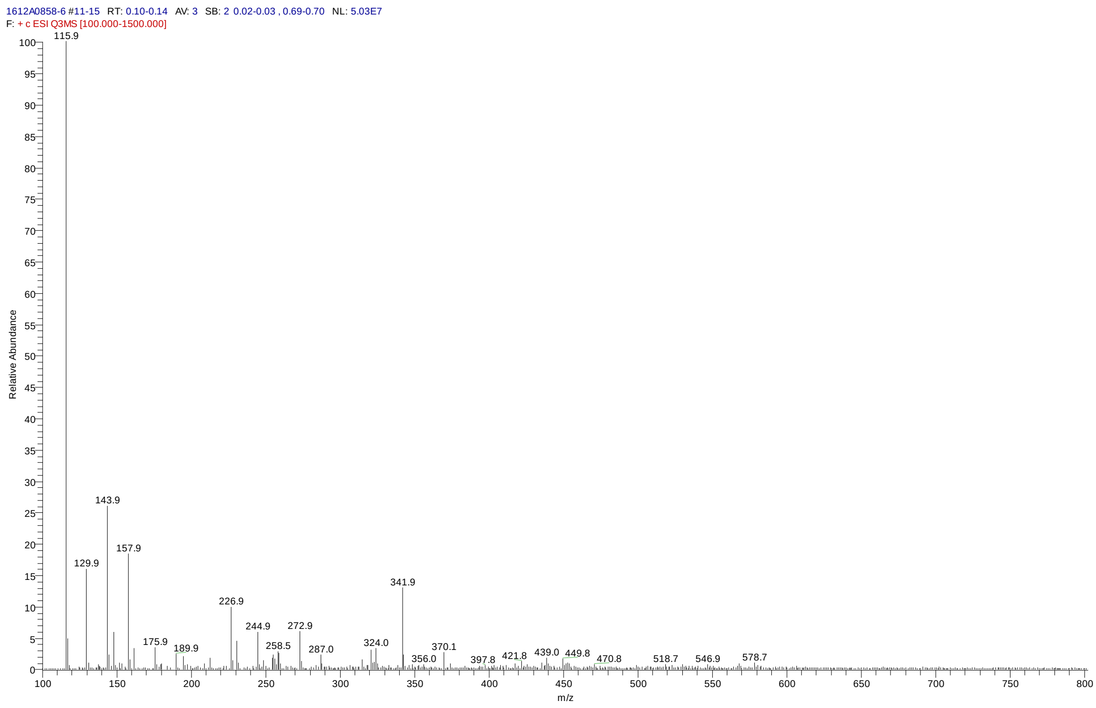

Supplementary Fig. S12 Mass spectral analysis of amino acid conjugate 109c.

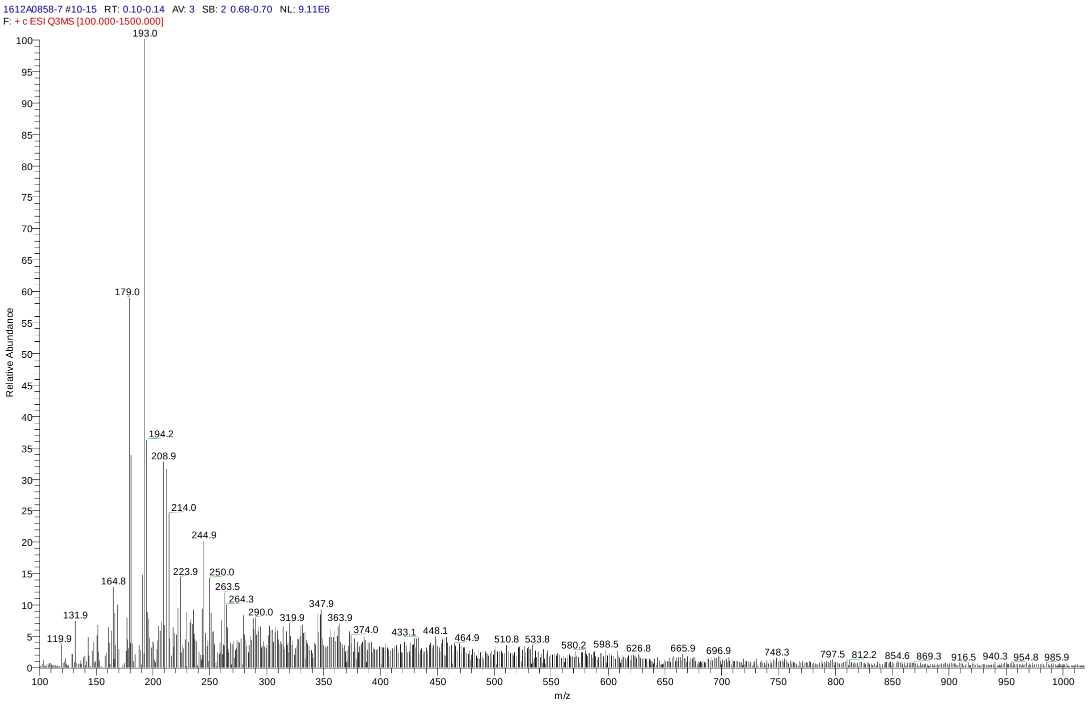

Supplementary Fig. S13 Mass spectral analysis of amino acid conjugate 112a.

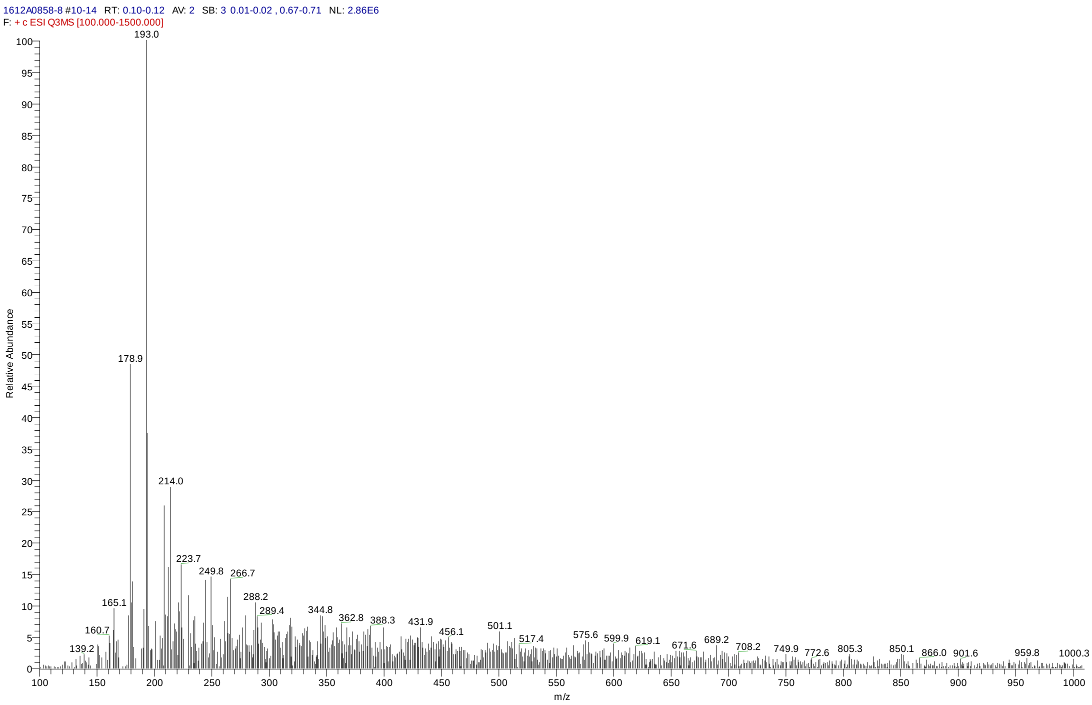

Supplementary Fig. S14 Mass spectral analysis of amino acid conjugate 112b.

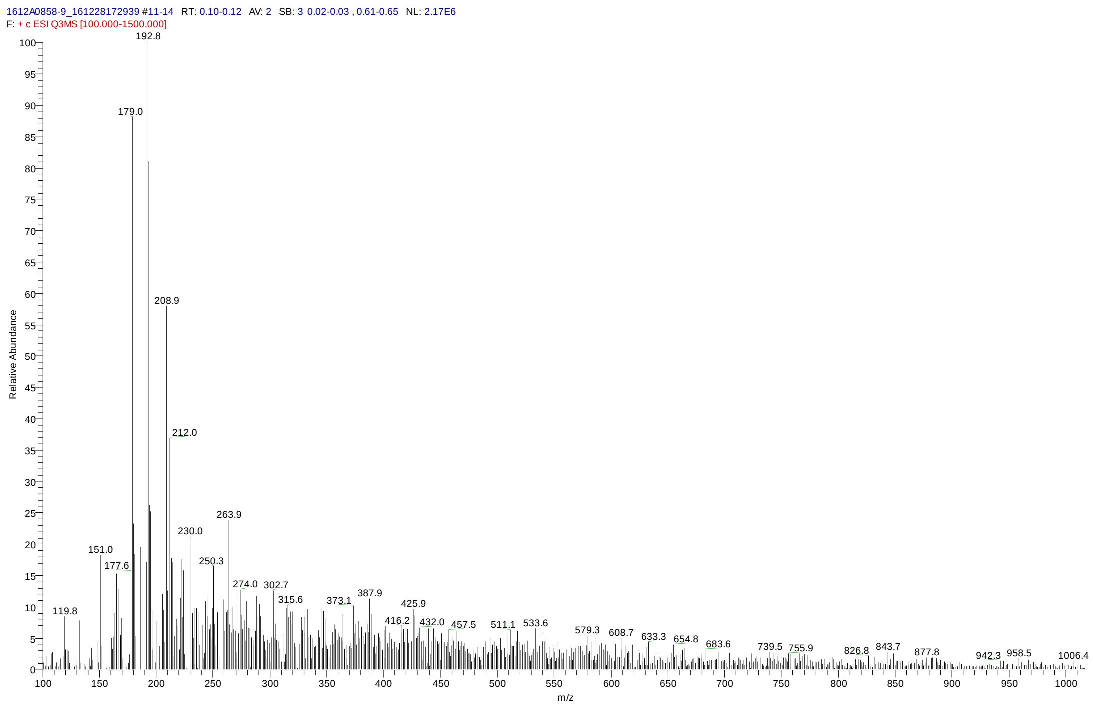

Supplementary Fig. S15 Mass spectral analysis of amino acid conjugate 112c.

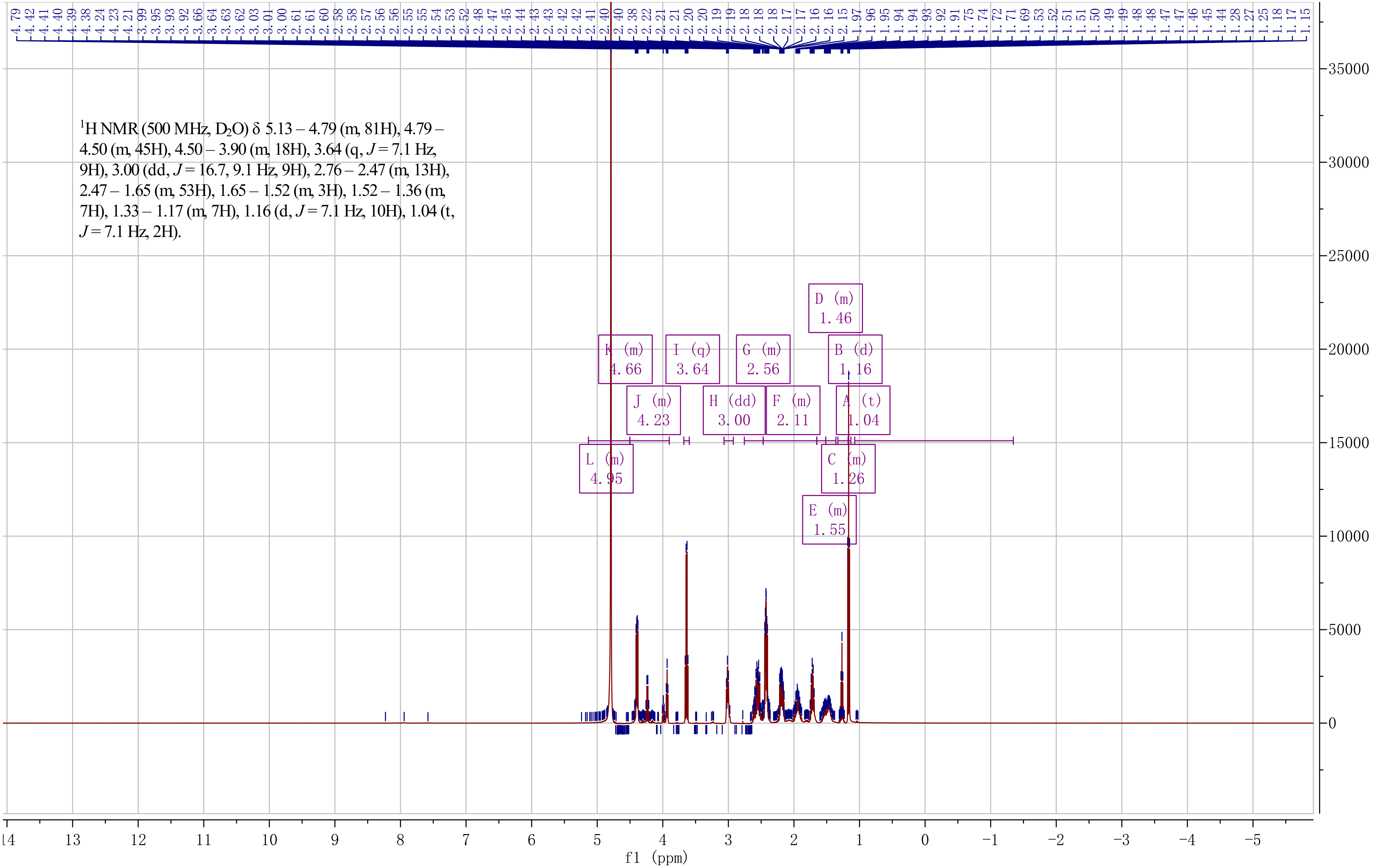

Supplementary Fig. S16 ^{1}H NMR of Conjugate 107a

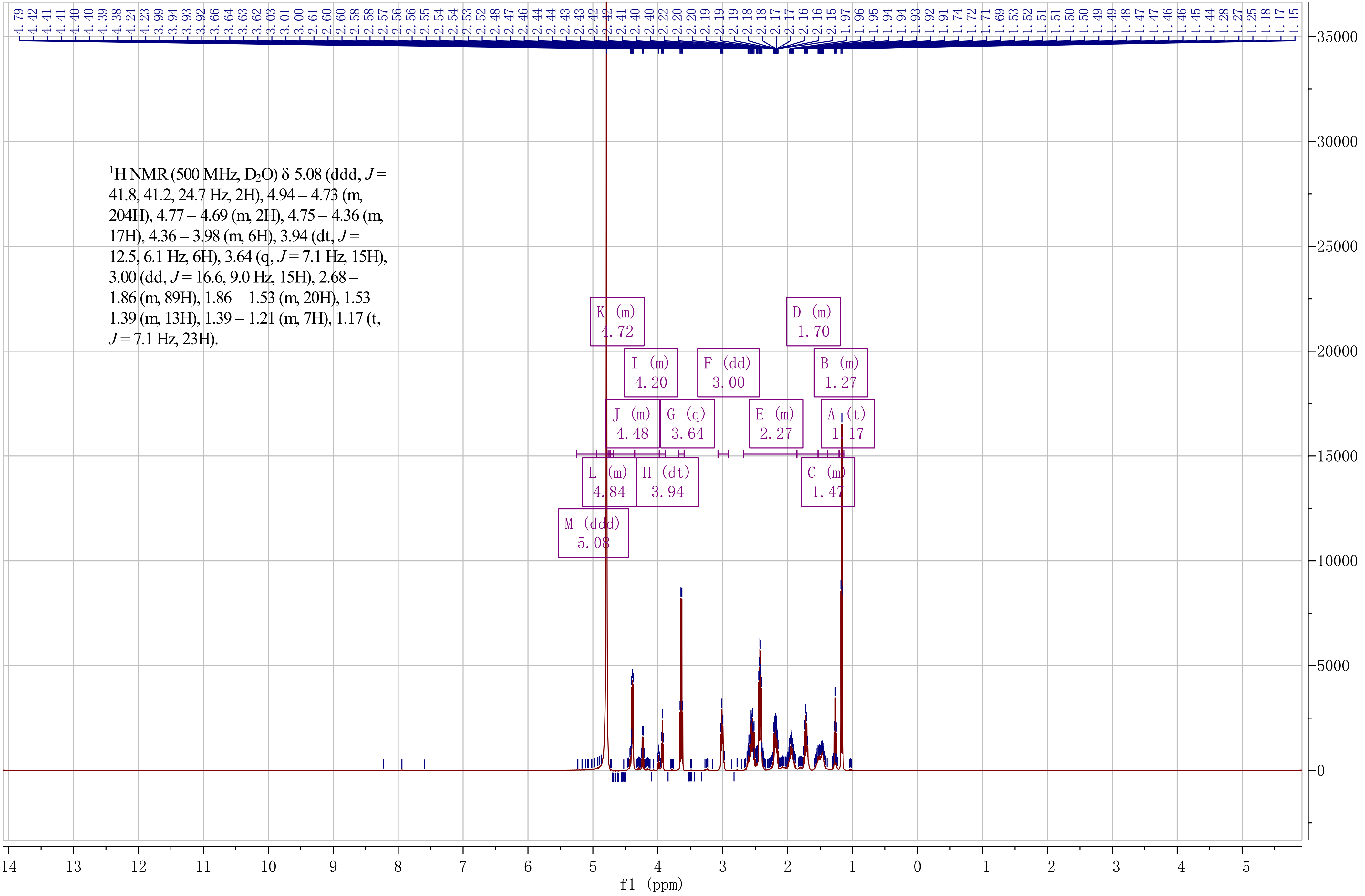

Supplementary Fig. S17 ^{1}H NMR of Conjugate 107b

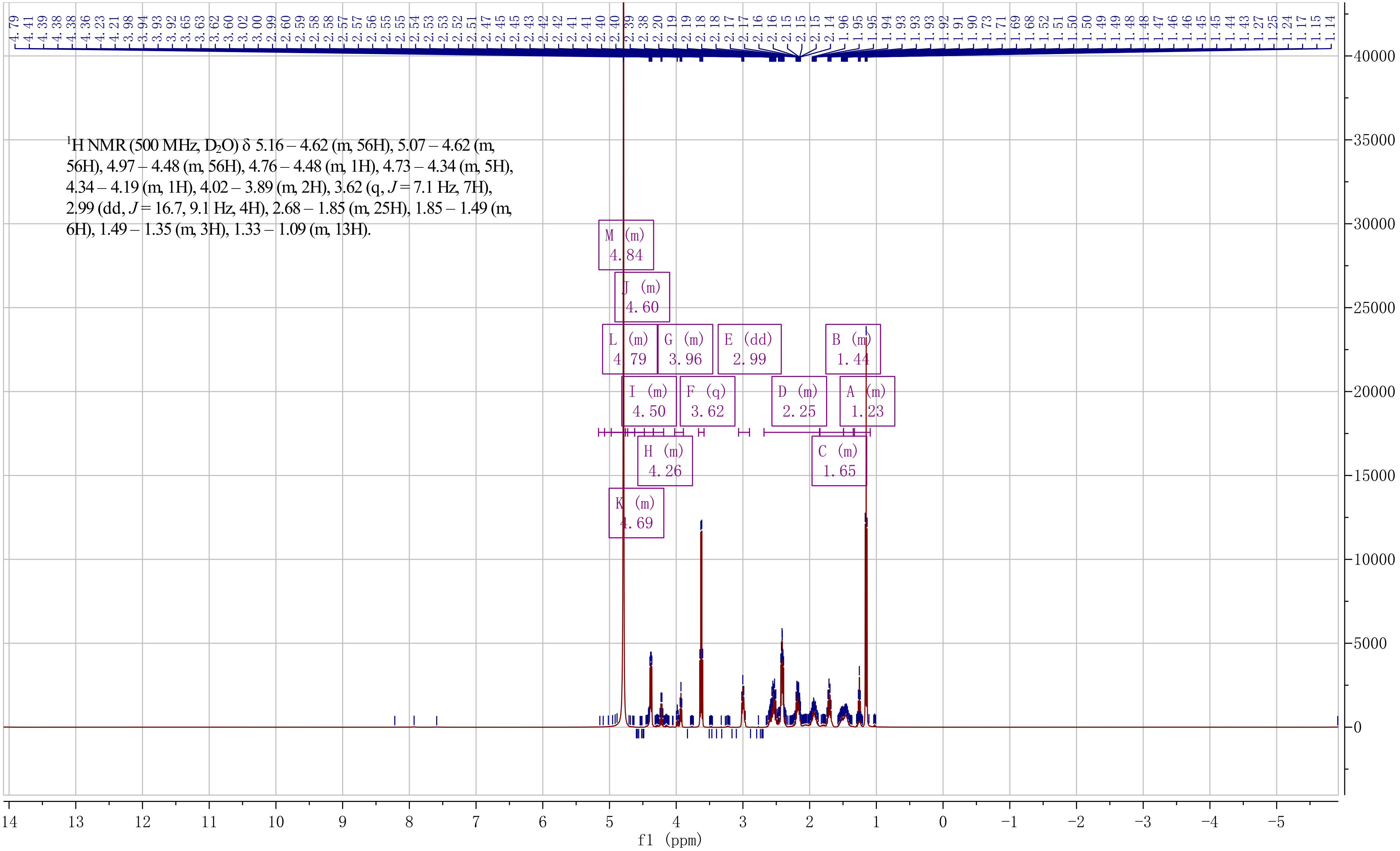

Supplementary Fig. S18 ^{1}H NMR of Conjugate 107c

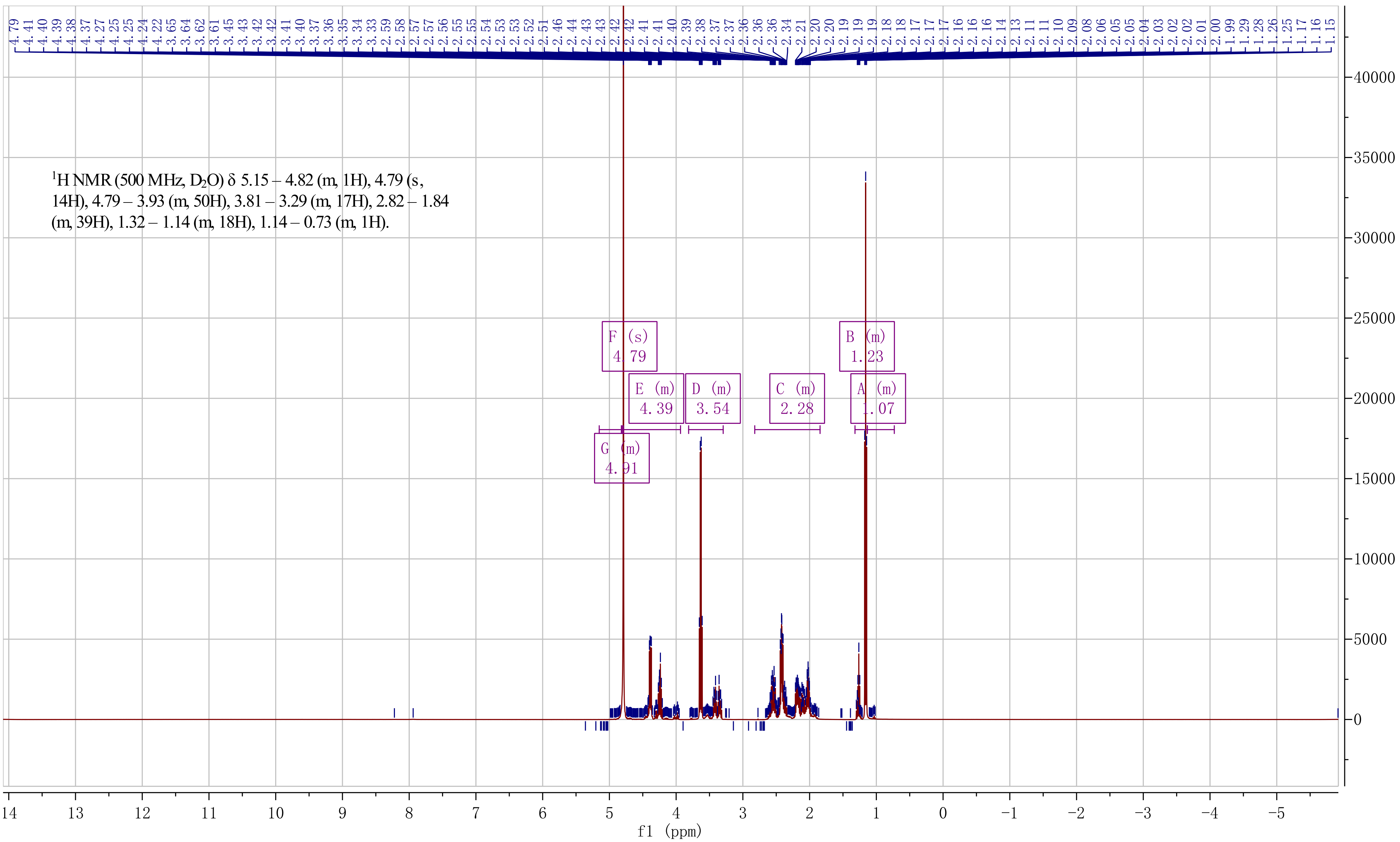

Supplementary Fig. S19 ^{1}H NMR of Conjugate 109a

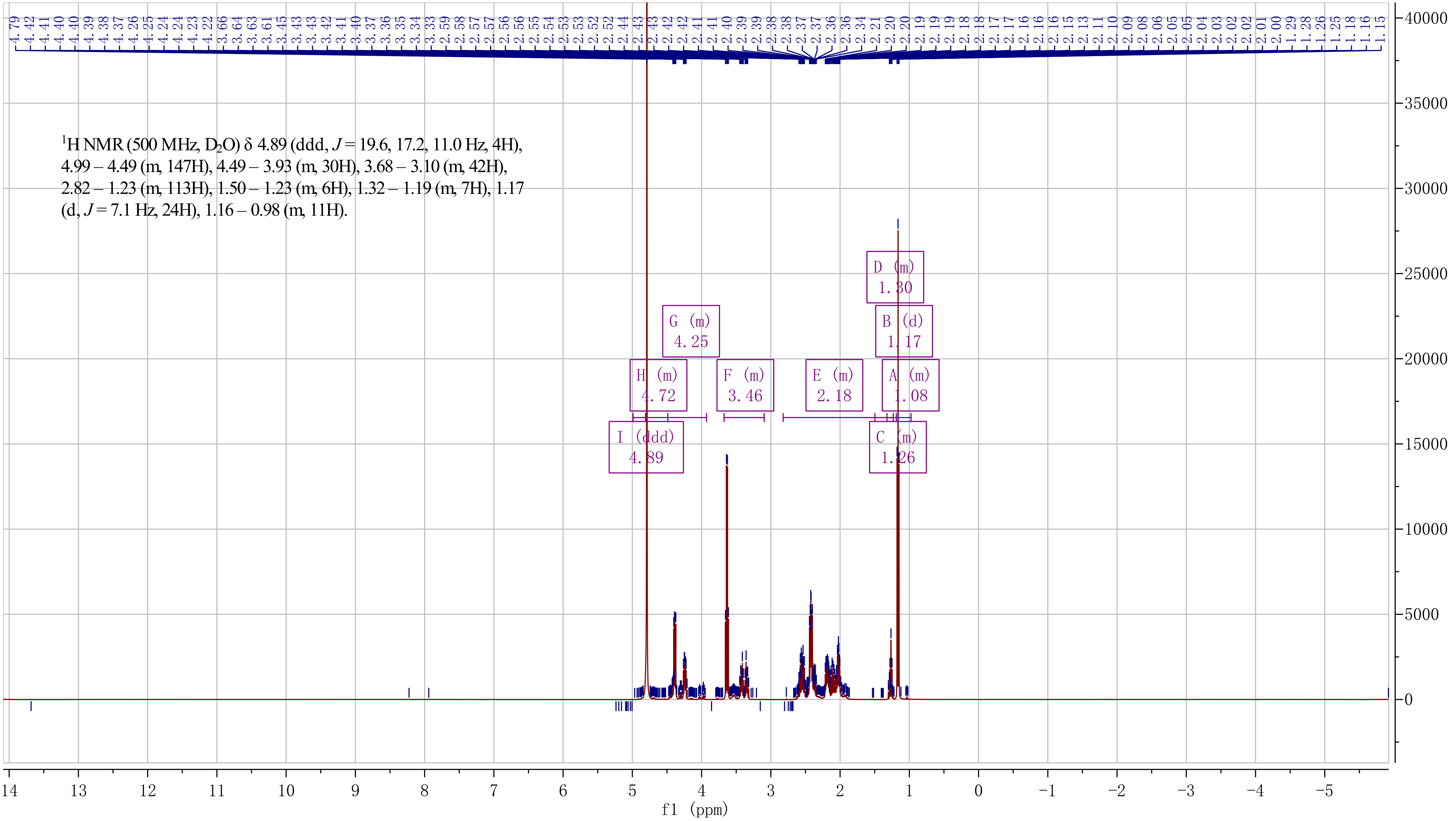

Supplementary Fig. S20 ^{1}H NMR of Conjugate 109b

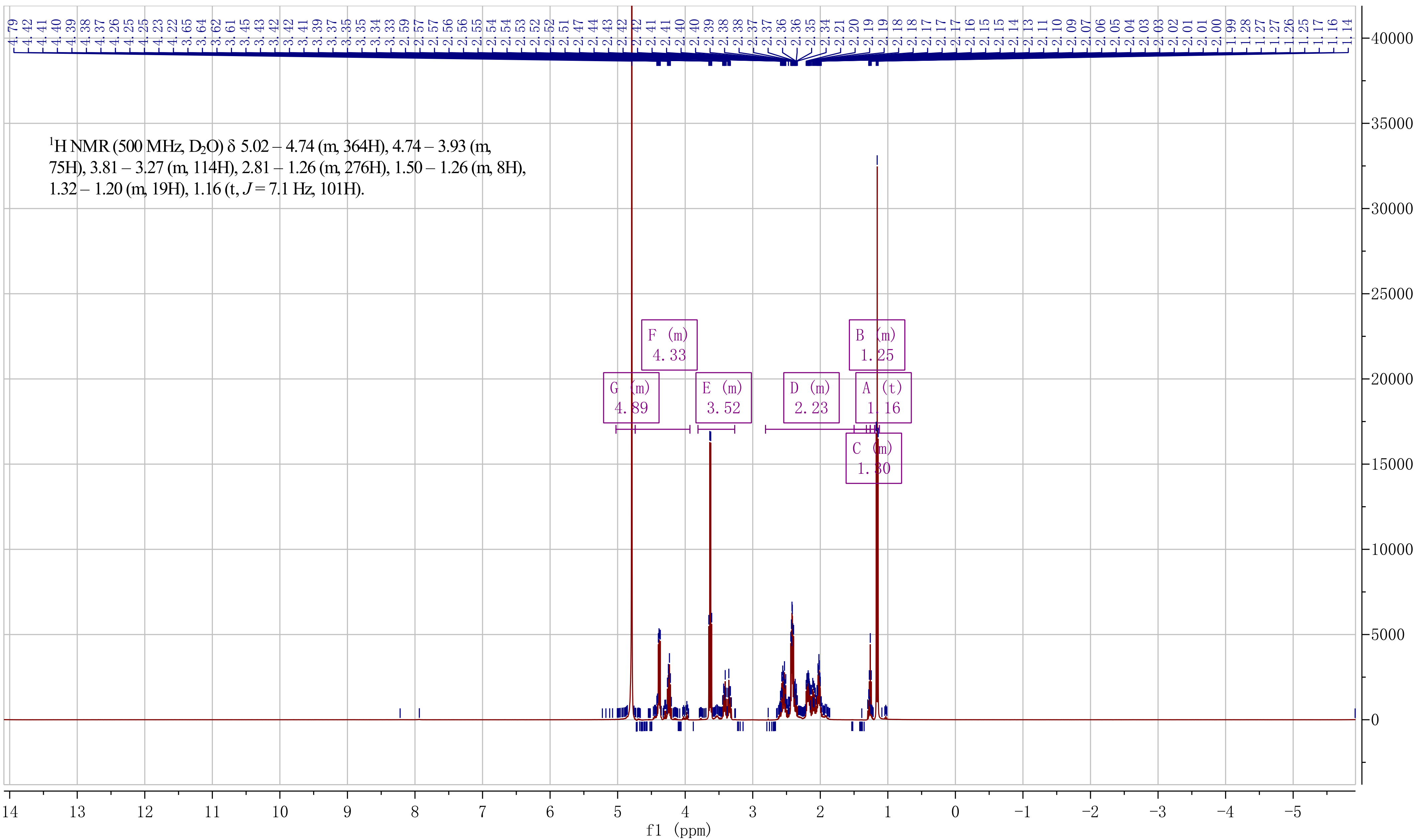

Supplementary Fig. S21 ^{1}H NMR of Conjugate 109c

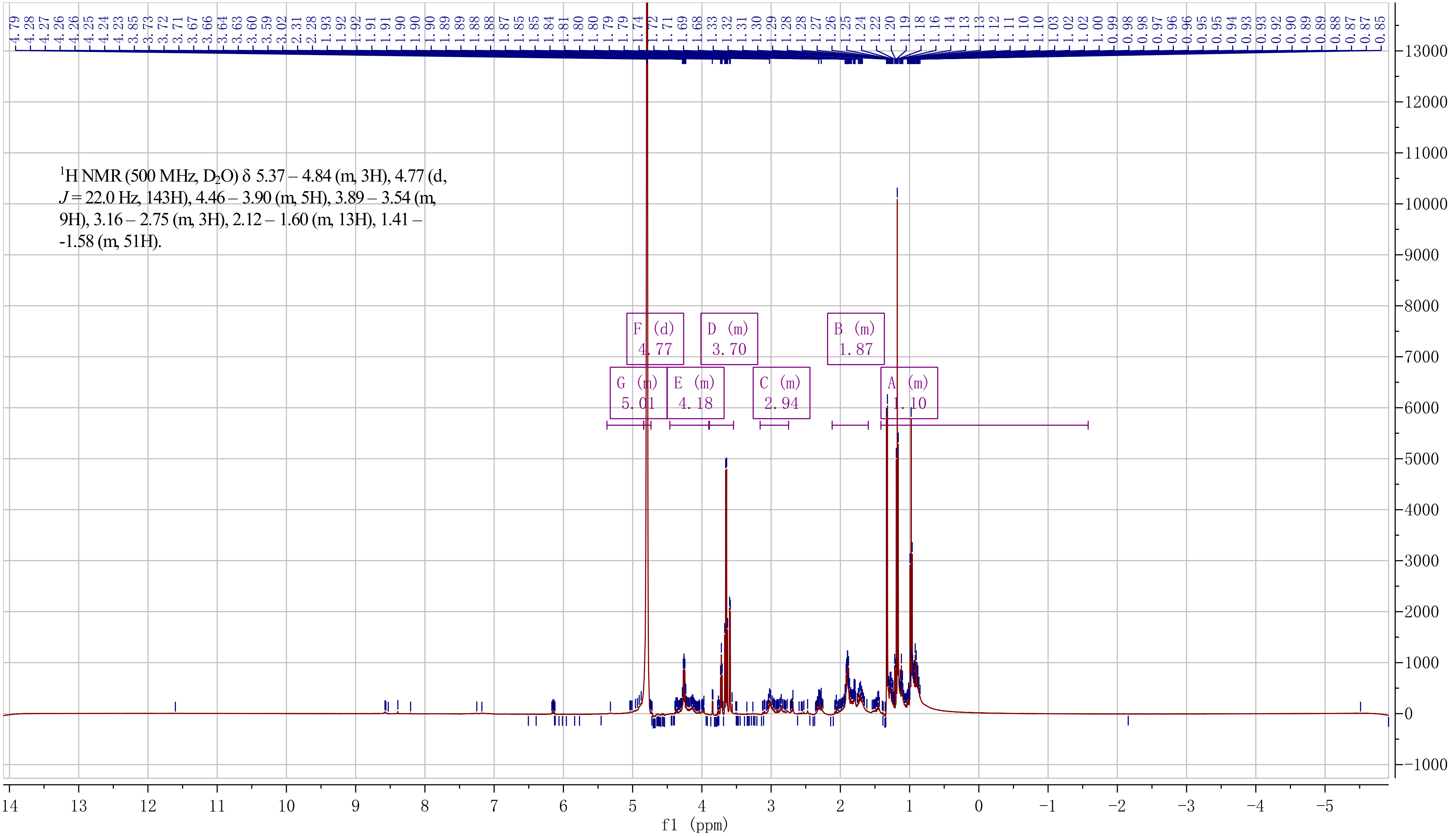

Supplementary Fig. S22 ^{1}H NMR of Conjugate 112a

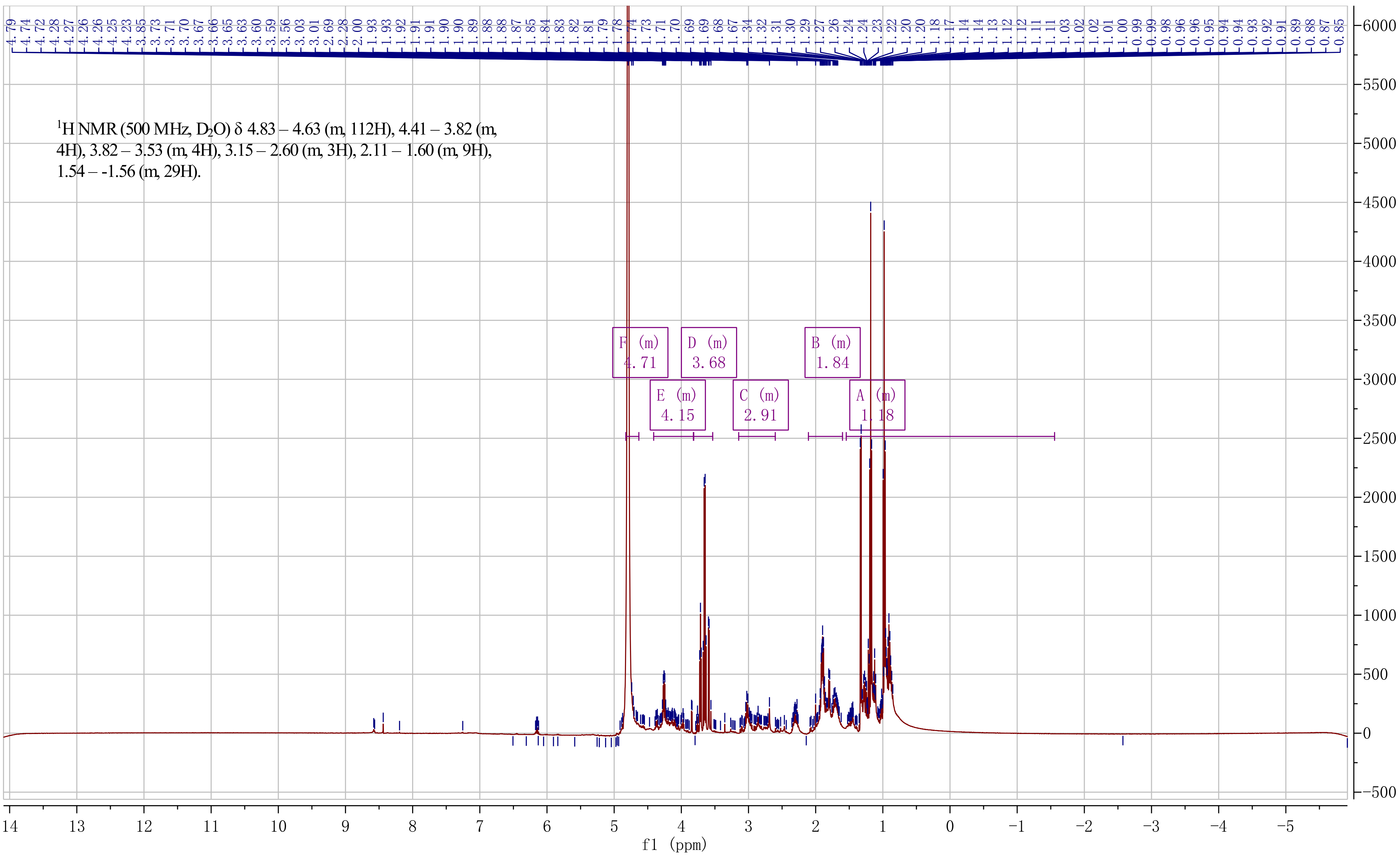

Supplementary Fig. S23 ^{1}H NMR of Conjugate 112b

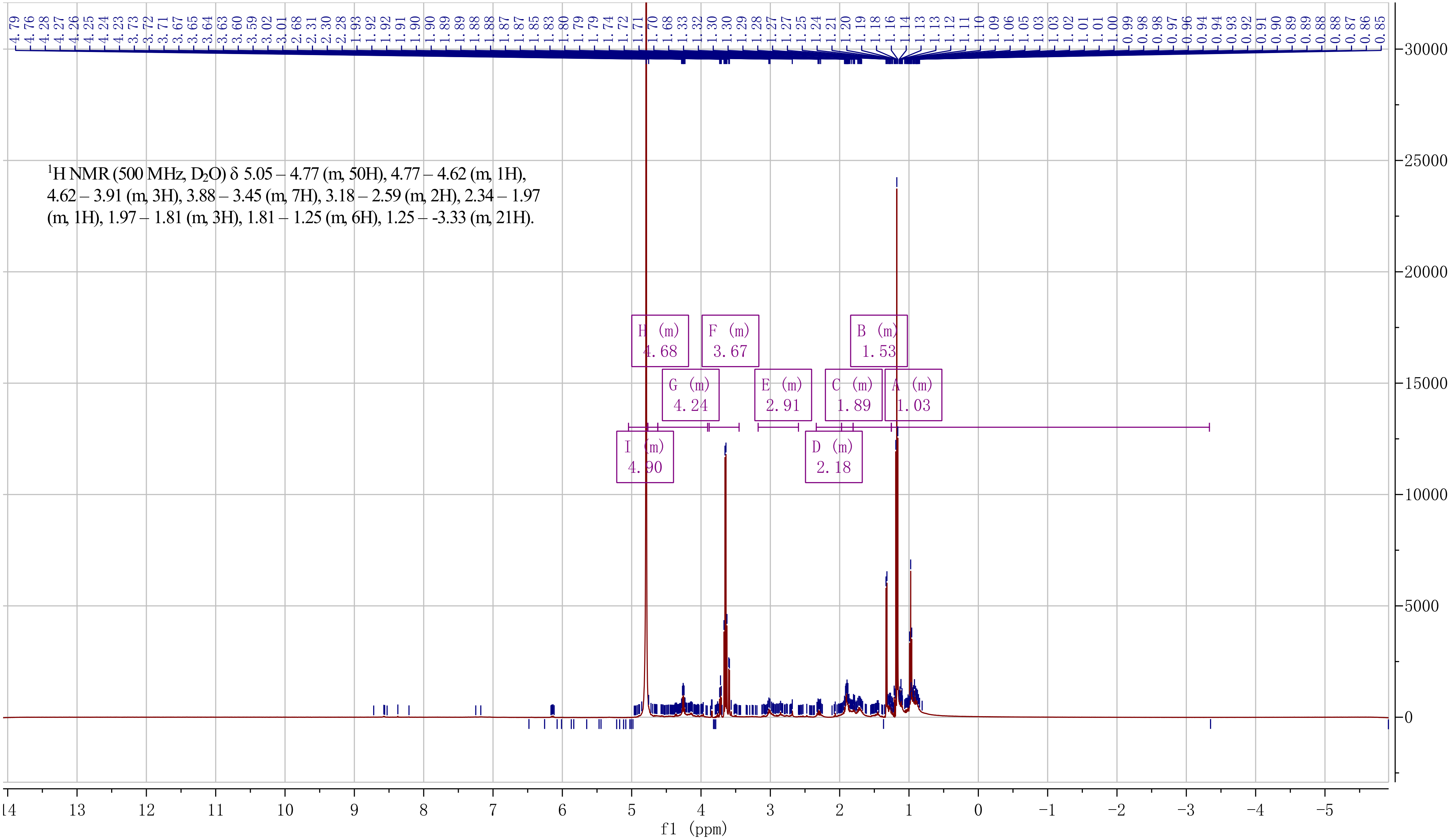

Supplementary Fig. S24 ^{1}H NMR of Conjugate 112c

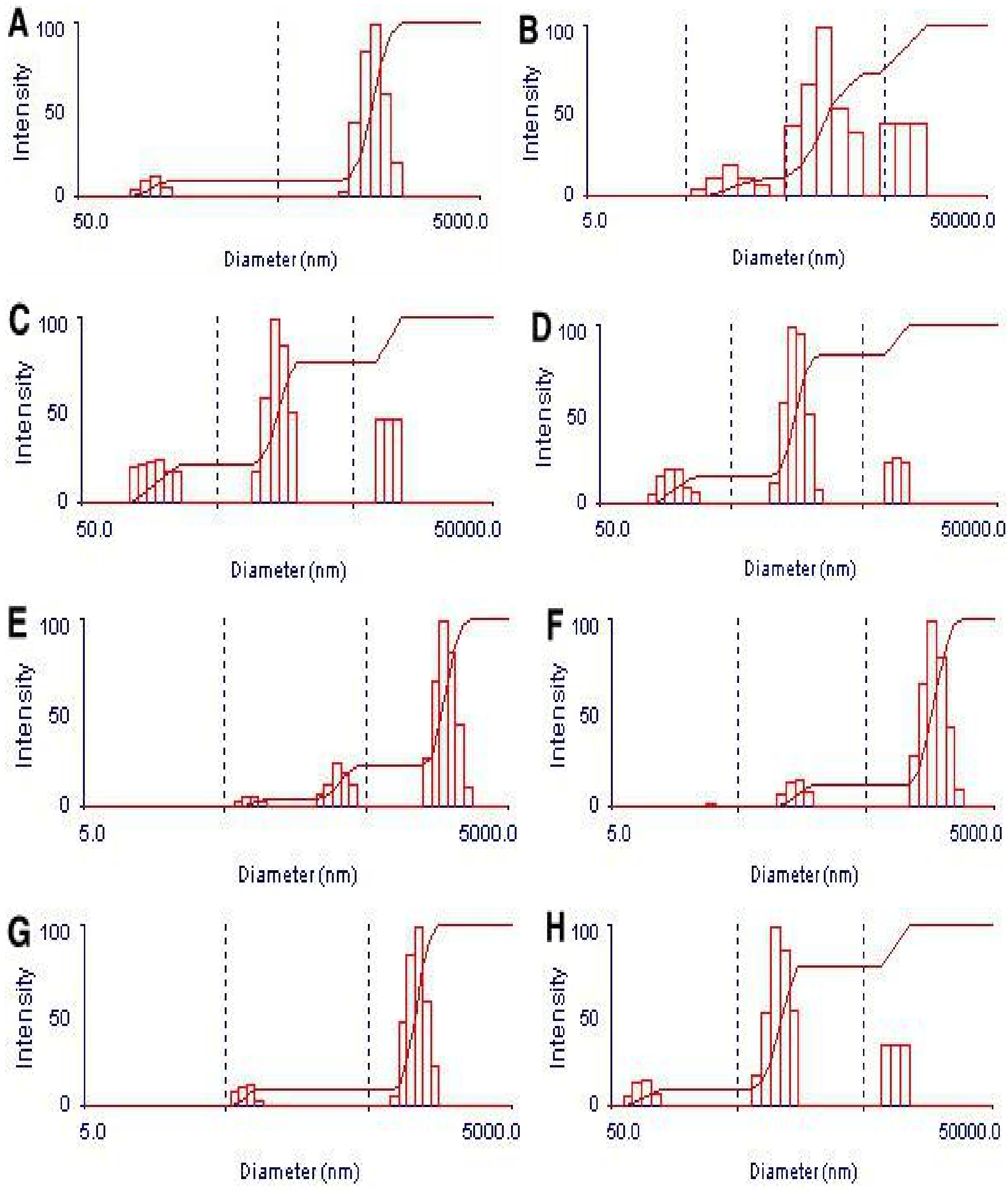

Supplementary Fig. S25 Particle sizes determined by Dynamic Light Scattering. Conjugate 107a (A-D), 109a (E-H). Control conjugate 112 was not determined due to insufficient solubility.

Supplementary Table S1. List of recipes for antibacterial conjugates*

No.	Recipe (w:w)	concentration	pH values of LSLP media with conjugates
16	L-Glu:L-Thr=2:1	0.5mg/ml	4.38
		0.25mg/ml	5.27
17	L-Leu:L-Lys HCl=1:8	0.5mg/ml	5.10
		0.25mg/ml	5.81
18	L-Leu:L-Lys HCl=1:8<br>Sunflower seed oil	0.5mg/ml	5.11
		0.25mg/ml	5.85
19	L-Leu:L-Lys HCl=1:8<br>Corn oil	0.5mg/ml	5.22
		0.25mg/ml	5.83
20	L-Leu:L-Lys HCl=1:8<br>Sesame oil	0.5mg/ml	5.29
		0.25mg/ml	6.02
33	L-Leu:L-Lys HCl=40:1	0.5mg/ml	5.64
		0.25mg/ml	6.20
40	L-Lys HCl	0.5mg/ml	4.91
		0.25mg/ml	5.91
50	L-Asp:L-Arg=1:1	0.5mg/ml	5.74
		0.25mg/ml	6.27
51	L-Asp:L-Arg=2:1	0.5mg/ml	4.82
		0.25mg/ml	5.48
52	L-Asp:L-Arg=4:1	0.5mg/ml	4.82
		0.25mg/ml	5.72
53	L-Asp:L-Lys=1:1	0.5mg/ml	4.80
		0.25mg/ml	5.47
54	L-Asp:L-Lys=2:1	0.5mg/ml	4.86
		0.25mg/ml	5.76
55	L-Asp:L-Lys=4:1	0.5mg/ml	5.31
		0.25mg/ml	5.95
57	L-Asp:L-His=2:1	0.5mg/ml	4.72
		0.25mg/ml	5.56
58	L-Asp:L-His=4:1	0.5mg/ml	4.39
		0.25mg/ml	5.07
59	L-Asp:L-Pro=1:1	0.5mg/ml	4.43
		0.25mg/ml	5.14
60	L-Asp:L-Pro=2:1	0.5mg/ml	5.21
		0.25mg/ml	5.83
62	L-Glu:L-Arg=1:1	0.5mg/ml	4.86
		0.25mg/ml	5.69

63	L-Glu:L-Arg=2:1	0.5mg/ml	4.36
		0.25mg/ml	4.94
64	L-Glu:L-Arg=4:1	0.5mg/ml	4.24
		0.25mg/ml	4.92
65	L-Glu:L-Lys HCl=1:1	0.5mg/ml	4.64
		0.25mg/ml	5.41
66	L-Glu:L-Lys HCl=2:1	0.5mg/ml	4.31
		0.25mg/ml	5.18
67	L-Glu:L-Lys HCl=4:1	0.5mg/ml	4.39
		0.25mg/ml	4.99
68	L-Glu:L-His=1:1	0.5mg/ml	4.31
		0.25mg/ml	4.96
69	L-Glu:L-His=2:1	0.5mg/ml	4.39
		0.25mg/ml	5.24
72	L-Glu:L-Pro=2:1	0.5mg/ml	4.24
		0.25mg/ml	5.12
73	L-Glu:L-Pro=4:1	0.5mg/ml	4.24
		0.25mg/ml	4.94
74	L-Lys HCl:L-Leu=4:1	0.5mg/ml	5.15
		0.25mg/ml	6.00
90	L-Lys HCl:L-Ser=4:1	0.5mg/ml	5.43
		0.25mg/ml	6.16
91	L-Lys HCl:L-Ser=8:1	0.5mg/ml	5.36
		0.25mg/ml	6.16
92	L-Lys HCl:L-Asp:L-Thr=1:1:8	0.5mg/ml	6.01
		0.25mg/ml	6.32
101	D-Glu:L-Thr=2:1	0.25 mg/ml	5.38
		0.125 mg/ml	5.83
102	D-Asp:DL-Arg=4:1	0.25 mg/ml	6.80
		0.125 mg/ml	6.85
103	D-Asp:D-Lys HCl=2:1	0.25 mg/ml	5.58
		0.125 mg/ml	5.91
104	D-Asp:LHis=2:1	0.25 mg/ml	5.56
		0.125 mg/ml	5.80
105	D-Asp:L-Pro=1:1	0.25 mg/ml	5.17
		0.125 mg/ml	5.52
106	D-Glu:DL-Arg =2:1	0.25 mg/ml	5.54
		0.125 mg/ml	5.90
107	D-Glu:D-Lys HCl=2:1	0.25 mg/ml	4.82

		0.125 mg/ml	5.38
108	D-Glu:L-His=2:1	0.25 mg/ml	5.35
		0.125 mg/ml	5.72
109	D-Glu:L-Pro=2:1	0.25 mg/ml	5.55
		0.125 mg/ml	5.89
110	D-Lys HCl:L-Ser=4:1	0.25 mg/ml	6.01
		0.125 mg/ml	6.30
111	D-Lys HCl:L-Ser=8:1	0.25 mg/ml	5.98
		0.125 mg/ml	6.30
112	D-Lys HCl:D-Asp:L-Thr=1:1:8	0.25 mg/ml	6.14
		0.125 mg/ml	6.45
113	D-Glu:L-Thr=2:1	0.25 mg/ml	5.33
		0.125 mg/ml	5.83
114	D-Asp:DL-Arg=4:1	0.25 mg/ml	5.94
		0.125 mg/ml	6.26
115	D-Asp:D-Lys HCl=2:1	0.25 mg/ml	5.78
		0.125 mg/ml	6.14
116	D-Asp:LHis=2:1	0.25 mg/ml	5.52
		0.125 mg/ml	5.87
117	D-Asp:L-Pro=1:1	0.25 mg/ml	5.48
		0.125 mg/ml	5.90
118	D-Glu:DL-Arg =2:1	0.25 mg/ml	5.32
		0.125 mg/ml	5.76
119	D-Glu:D-Lys HCl=2:1	0.25 mg/ml	5.03
		0.125 mg/ml	5.57
120	D-Glu:L-His=2:1	0.25 mg/ml	4.86
		0.125 mg/ml	5.31
121	D-Glu:L-Pro=2:1	0.25 mg/ml	5.05
		0.125 mg/ml	5.70
122	D-Lys HCl:L-Ser=4:1	0.25 mg/ml	5.92
		0.125 mg/ml	6.15
123	D-Lys HCl:L-Ser=8:1	0.25 mg/ml	5.74
		0.125 mg/ml	6.17
124	D-Lys HCl :D-Asp:L-Thr=1:1:8	0.25 mg/ml	6.22
		0.125 mg/ml	6.37

* Peanut oil was used for all heat conjugations unless specified. Conjugates 113-124 were synthesized with rapeseed oil.

Supplementary Table S2. Counterparts of conjugates with D-amino acid as part of starting materials.

Conjugate using peanut oil	Conjugate using rapeseed oil	Corresponding conjugate with all L-amino acid starting materials
101	113	16
102	114	52
103	115	54
104	116	57
105	117	59
106	118	63
107	119	66
108	120	69
109	121	72
110	122	90
111	123	91
112	124	92

Supplementary Table S3. ^{1}H NMR of the most active and control antimicrobial conjugates with 3 replicates for each recipe.

Conjugates	^{1}H NMR (500 MHz, D_2O)
107a	δ 5.06 – 4.82 (m, 5H), 4.81 – 4.50 (m, 263H), 4.50 – 3.94 (m, 35H), 3.93 (d, *J* = 6.2 Hz, 5H), 3.64 (q, *J* = 7.1 Hz, 19H), 3.00 (dd, *J* = 16.7, 9.1 Hz, 20H), 2.76 – 1.86 (m, 122H), 1.86 – 1.52 (m, 29H), 1.52 – 1.36 (m, 15H), 1.33 – 1.16 (m, 31H), 1.15 (s, 7H).
107b	δ 5.08 (ddd, *J* = 41.8, 41.2, 24.7 Hz, 2H), 4.94 – 4.73 (m, 204H), 4.77 – 4.69 (m, 2H), 4.75 – 4.36 (m, 17H), 4.36 – 3.98 (m, 6H), 3.94 (dt, *J* = 12.5, 6.1 Hz, 6H), 3.64 (q, *J* = 7.1 Hz, 15H), 3.00 (dd, *J* = 16.6, 9.0 Hz, 15H), 2.68 – 1.86 (m, 89H), 1.86 – 1.53 (m, 20H), 1.53 – 1.39 (m, 13H), 1.39 – 1.21 (m, 7H), 1.17 (t, *J* = 7.1 Hz, 23H).
107c	δ 5.16 – 4.62 (m, 56H), 5.07 – 4.62 (m, 56H), 4.97 – 4.48 (m, 56H), 4.76 – 4.48 (m, 1H), 4.73 – 4.34 (m, 5H), 4.34 – 4.19 (m, 1H), 4.02 – 3.89 (m, 2H), 3.62 (q, *J* = 7.1 Hz, 7H), 2.99 (dd, *J* = 16.7, 9.1 Hz, 4H), 2.68 – 1.85 (m, 25H), 1.85 – 1.49 (m, 6H), 1.49 – 1.35 (m, 3H), 1.33 – 1.09 (m, 13H).
109a	δ 5.15 – 4.82 (m, 1H), 4.79 (s, 14H), 4.79 – 3.93 (m, 50H), 3.81 – 3.29 (m, 17H), 2.82 – 1.84 (m, 39H), 1.32 – 1.14 (m, 18H), 1.14 – 0.73 (m, 1H).
109b	δ 4.89 (ddd, *J* = 19.6, 17.2, 11.0 Hz, 4H), 4.99 – 4.49 (m, 147H), 4.49 – 3.93 (m, 30H), 3.68 – 3.10 (m, 42H), 2.82 – 1.23 (m, 113H), 1.50 – 1.23 (m, 6H), 1.32 – 1.19 (m, 7H), 1.17 (d, *J* = 7.1 Hz, 24H), 1.16 – 0.98 (m, 11H).
109c	δ 5.02 – 4.74 (m, 364H), 4.74 – 3.93 (m, 75H), 3.81 – 3.27 (m, 114H), 2.81 – 1.26 (m, 276H), 1.50 – 1.26 (m, 8H), 1.32 – 1.20 (m, 19H), 1.16 (t, *J* = 7.1 Hz, 101H).
112a	δ 5.37 – 4.84 (m, 3H), 4.77 (d, *J* = 22.0 Hz, 143H), 4.46 – 3.90 (m, 5H), 3.89 – 3.54 (m, 9H), 3.16 – 2.75 (m, 3H), 2.12 – 1.60 (m, 13H), 1.41 – -1.58 (m, 51H).
112b	δ 4.83 – 4.63 (m, 112H), 4.41 – 3.82 (m, 4H), 3.82 – 3.53 (m, 4H), 3.15 – 2.60 (m, 3H), 2.11 – 1.60 (m, 9H), 1.54 – -1.56 (m, 29H).
112c	δ 5.05 – 4.77 (m, 50H), 4.77 – 4.62 (m, 1H), 4.62 – 3.91 (m, 3H), 3.88 – 3.45 (m, 7H), 3.18 – 2.59 (m, 2H), 2.34 – 1.97 (m, 1H), 1.97 – 1.81 (m, 3H), 1.81 – 1.25 (m, 6H), 1.25 – -3.33 (m, 21H).

Supplementary Table S4. Extraction with n-hexane and chloroform, and hydrolysis of antibacterial conjugates with 3 replicates for each recipe.

Conjugate no.	Starting material (mg) for extraction	n-hexane extractable weight (% recovery, pH)*	Chloroform extractable weight (% recovery, pH)*	Residuals' weight after n-hexane and chloroform extraction (pH)	Starting material (mg) for alkaline hydrolysis	n-hexane extractable weight (% recovery) (post-hydrolysis)	Amino acid content at 570 nm (%) (post-hydrolysis)	Amino acid content at 440 nm (%) (post-hydrolysis)
107a	30.0	0.5 (1.68%, 6.05)	1.0 (3.36%, 5.73)	54.7 (3.71)	30.0	1.29 (4.3%)	6.83 (22.77 %)	9.16 (30.52 %)
107b	30.0	0.5 (1.68%, 5.80)	1.0 (3.36%, 5.59)	58.0 (3.85)	30.0	1.50 (5.0%)	5.66 (18.88 %)	8.34 (27.80 %)
107c	30.0	0.5 (1.66%, 6.05)	0.8 (2.65%, 5.79)	55.7 (3.61)	30.0	1.50 (5.0%)	5.67 (18.89 %)	8.21 (27.35 %)
109a	30.0	0.4 (1.32%, 6.03)	0.9 (2.96%, 5.15)	55.9 (3.93)	30.0	1.11 (3.7%)	5.52 (18.39 %)	10.38 (34.58 %)
109b	30.0	0.5 (1.69%, 5.97)	0.4 (1.36%, 5.23)	56.5 (3.48)	30.0	1.20 (4.0%)	6.13 (20.43 %)	11.33 (37.77 %)
109c	30.0	0.6 (1.95%, 6.04)	1.3 (4.23%, 5.55)	56.8 (3.74)	30.0	0.96 (3.2%)	6.43 (21.42 %)	11.79 (39.31 %)
112a	30.0	1.2 (4.04%, 6.04)	1.8 (6.06%, 5.47)	55.5 (4.94)	30.0	1.74 (5.8%)	2.18 (7.26%)	1.69 (5.62%)
112b	30.0	1.7 (5.73%, 6.16)	1.9 (6.33%, 5.23)	53.3 (4.84)	30.0	1.44 (4.8%)	2.00 (6.67%)	2.28 (7.60%)
112c	30.0	1.9 (6.33%, 5.87)	1.5 (5.00%, 5.49)	53.7 (4.49)	30.0	1.98 (6.6%)	3.52 (11.74 %)	2.75 (9.17%)

*N-hexane or chloroform extractable constituents showed poor or modest antibacterial activities according to MIC assays.
